# Weight loss strategies, weight change, and type 2 diabetes in US health professionals: A cohort study

**DOI:** 10.1371/journal.pmed.1004094

**Published:** 2022-09-27

**Authors:** Keyi Si, Yang Hu, Molin Wang, Caroline M. Apovian, Jorge E. Chavarro, Qi Sun

**Affiliations:** 1 Department of Nutrition, Harvard T.H. Chan School of Public Health, Boston, Massachusetts, United States of America; 2 Department of Epidemiology, Harvard T.H. Chan School of Public Health, Boston, Massachusetts, United States of America; 3 Department of Biostatistics, Harvard T.H. Chan School of Public Health, Boston, Massachusetts, United States of America; 4 Division of Endocrinology, Diabetes, and Hypertension, Department of Medicine, Brigham and Women’s Hospital and Harvard Medical School, Boston, Massachusetts, United States of America; 5 Channing Division of Network Medicine, Department of Medicine, Brigham and Women’s Hospital and Harvard Medical School, Boston, Massachusetts, United States of America; Carolina Population Center, UNITED STATES

## Abstract

**Background:**

Weight loss is crucial for disease prevention among individuals with overweight or obesity. This study aimed to examine associations of weight loss strategies (WLSs) with weight change and type 2 diabetes (T2D) risk among US health professionals.

**Methods and findings:**

This study included 93,110 participants (24 to 60 years old; 11.6% male) from the Nurses’ Health Study (NHS), NHSII, and Health Professionals Follow-Up Study (HPFS) cohorts who were free of T2D, cardiovascular disease, and cancer at baseline (1988 for NHS/HPFS and 1989 for NHSII) for analyses of weight change and 104,180 (24 to 78 years old; 14.2% male) for T2D risk assessment. WLSs used to achieve an intentional weight loss of 4.5+ kg were collected in 1992 (NHS/HPFS)/1993 (NHSII) and grouped into 7 mutually exclusive categories, including low-calorie diet, exercise, low-calorie diet and exercise, fasting, commercial weight loss program (CWLP), diet pills, and FCP (selected at least 2 methods from fasting, CWLP, and pill). The reference group was participants who did not attempt to lose weight. Generalized estimating equations and Cox regression were applied to estimate up to 10-year weight change trajectory and incident T2D risk through 2016 (NHS/HPFS)/2017 (NHSII), respectively.

The associations of WLSs with weight change and T2D risk were differential by baseline body weight (P_interaction_ < 0.01). Among individuals with obesity, all WLSs tended to associate with less weight gain [ranging from −4.2% (95% confidence interval (CI), −5.1% to −3.2%; *P* < 0.001) for exercise to −0.3% (−1.2% to 0.7%; *P* > 0.99) for FCP] and a lower T2D risk [hazard ratios (HRs) ranging from 0.79 (0.66 to 0.95; *P* = 0.04) for exercise to 0.87 (0.66 to 1.13; *P* = 0.30) for pill]. Such a pattern was less clear among overweight individuals: the difference of weight change varied from −2.5% (−3.0% to −2.1%; *P* < 0.001) for exercise to 2.0% (1.3% to 2.7%; *P* < 0.001) for FCP, and HRs of T2D varied from 0.91 (0.77 to 1.07; *P* = 0.29) for exercise to 1.42 (1.11 to 1.81; *P* = 0.02) for pill. The pattern was further inverted among lean individuals in that weight change ranged from −0.4% (−0.6% to −0.1%; *P* = 0.02) for exercise to 3.7% (3.1% to 4.3%; *P* < 0.001) for FCP, and the HRs of T2D ranged from 1.09 (0.91 to 1.30; *P* = 0.33) for exercise to 1.54 (1.13 to 2.10; *P* = 0.008) for pill. Approximately 15.6% to 46.8% of the association between WLSs and the T2D risk was attributed to weight changes. This study was limited by a single assessment of WLSs, heterogeneity within each WLS, and potential misclassification of the timing of weight loss and weight regain.

**Conclusions:**

The current study showed that individuals with obesity who attempted to lose weight, regardless of the WLSs used, tended to gain less body weight and have a lower diabetes risk. In contrast, lean individuals who intentionally lost weight tended to gain more weight and have a higher diabetes risk. These data support the notion that intentional weight loss may not be beneficial for lean individuals and the use of WLSs for achieving weight loss shall be guided by medical indications only.

## Abstract 2

Keyi Si and colleagues investigate associations of weight loss strategies with weight gain and type 2 diabetes in health professionals in the US.

## Author summary

**Why was this study done?:**

- Weight control is one of the primary and effective strategies for the prevention and management of obesity and related chronic diseases.
- Trying to lose weight is common not only among overweight individuals or those with obesity, but also among lean individuals.
- Long-term weight change and risks of developing type 2 diabetes (T2D) following various weight loss strategies (WLSs) are understudied.

**What did the researchers do and find?:**

- Up to 10-year weight change and 24-year T2D risk were compared between individuals who lost 4.5+ kg (10+ lbs) through various WLSs (low-calorie diet, exercise, low-calorie diet and exercise, fasting, commercial weight loss program (CWLP), diet pills, or a combination of the last 3 strategies collectively named as FCP) and those who did not attempt to lose weight.
- Association of WLSs with weight change and T2D risk varied by baseline body mass index status: individuals with obesity who lost 4.5+ kg intentionally were likely to have less weight gain within 4 years since baseline and lower risk of T2D during 24 years of follow-up, regardless of WLS, but the beneficial associations were attenuated in individuals with overweight and even inverted in lean individuals.
- Of all WLSs, exercise was associated with the least weight gain and the lowest T2D risk among individuals with obesity.
- Approximately 15.6% to 46.8% of the association between WLSs and the T2D risk was attributable to body weight changes after weight loss attempts.

**What do these findings mean?:**

- Individuals with obesity may benefit from intentional weight loss regardless of the methods used to achieve the weight loss, whereas lean individuals may not enjoy the same health benefits from intentionally losing a significant amount of body weight.
- These data suggest that the use of weight loss methods for achieving weight loss should be guided by medical or health indications.

## Introduction

Obesity is one of the most common chronic conditions in the United States and globally. In 2017 to 2018, 42.4% of US adults were estimated to have obesity [1], which predisposed them to numerous chronic diseases, especially type 2 diabetes (T2D) [2]. As such, weight control is one of the primary and effective strategies for the prevention and management of chronic diseases with obesity-related etiology. On average, the risk of diabetes is estimated to reduce by 16% per kilogram weight loss in individuals with overweight/obesity and prediabetes [3]. In 2013 to 2016, 49.1% of US adults reported trying to lose weight, mainly through lifestyle modifications, such as exercise (62.9%) and dieting (62.9%) [4]. However, it is challenging to maintain weight loss, which is often accompanied by weight regain in the long run [5]. Meanwhile, a systematic review of 8 weight loss trials suggested that the weight regain trajectory in 3 to 5 years after the interventions varied by different weight loss strategies (WLSs) [6], which may thus exert differential impacts on the risk of developing obesity-related conditions, such as T2D. However, to our knowledge, no study has comprehensively examined multiple commonly practiced WLSs in relation to long-term weight change trajectories or T2D risk in free-living individuals who choose WLSs at will in observational study settings.

To fill the knowledge gaps, the current study aimed to investigate the association of common WLSs with weight change and T2D risk in 3 large-scale prospective cohorts of free-living US men and women. In addition, in light of the evidence that baseline body weight may modulate the benefits of weight loss [7–9], we also evaluated the associations of interest according to baseline body mass index (BMI) before weight loss attempts.

## Materials and methods

This study is reported as per the Strengthening the Reporting of Observational Studies in Epidemiology (STROBE) guideline (**S1 Checklist**). The study protocol was drafted prospectively in August 2019 (**S1 Text**).

### Study population

This study used data from the Health Professionals Follow-up Study (HPFS), the Nurses’ Health Study (NHS), and the NHSII. The HPFS began in 1986 and enrolled 51,529 male health professionals aged 40 to 76 years from 50 US states. The NHS recruited 121,701 female nurses aged 30 to 55 years from 11 states in 1976. The NHSII, initiated in 1989, included 116,429 female nurses aged 24 to 42 years from 14 states. Follow-up questionnaires were mailed to participants biennially since 1976 (NHS)/1986 (HPFS)/1989 (NHSII) to update lifestyle and medical information. Additional validated semiquantitative food frequency questionnaires were administered every 2 to 4 years thereafter to assess dietary intake. The response rates exceeded 90% in each cycle for 3 cohorts. Weight loss attempts within the last 4 years were self-reported in 1992 (NHS/HPFS)/1993 (NHSII). Since we did not know the exact time when the weight loss began in these 4 years, we considered 1988 for NHS/HPFS and 1989 for NHSII as study baseline.

For the analyses with T2D as the outcome, participants were excluded if they skipped the question of WLSs; if their weight loss was unintentional; if they reported a diagnosis of diabetes, cardiovascular disease, or cancer or deceased by 1992 (NHS/HPFS)/1993 (NHSII); if they only completed the 1992/1993 questionnaire; if they had missing information on the diagnosis date of T2D, age, or baseline BMI; or if they were pregnant at baseline (NHSII only). Other exclusions related to specific WLSs were listed in the next section. In analyses of weight change, the exclusion criteria were the same as those of T2D analyses, except that participants who only answered the 1992/1993 questionnaire remained if they provided valid body weight assessments in that year. Participants who did not report body weight since 1992/1993 or those aged 65+ years in 1992/1993 were further excluded. After the exclusions, 104,180 participants were included in the T2D analyses and 93,110 were considered in the weight change analyses (**S1 Fig**).

This study was approved by the institutional review boards of the Brigham and Women’s Hospital and the Harvard T.H. Chan School of Public Health, who deemed that the return of a complete self-administered questionnaire implied an informed consent. The last author vouched for the accuracy and completeness of the data and the analyses.

### Assessment of weight loss strategies

In the 1992 (NHS/HPFS) and 1993 (NHSII) questionnaires, participants were inquired about the amount (2.3 to 4.1 kg [5 to 9 lbs], 4.5 to 8.6 kg [10 to 19 lbs], 9.1 to 22.2 kg [20 to 49 lbs], 22.7+ kg [50+ lbs]) and frequency (0 times, 1 to 2 times, 3 to 4 times, 5 to 6 times, 7+ times) of intentional weight loss. Participants were asked to mark all the primary methods they had used to achieve the most recent weight loss of 4.5+ kg (10+ lbs) within the last 4 years, including the following possible responses: “did not lose 4.5+ kg,” “weight loss was unintentional (e.g., illness, unusual stress, depression),” “low-calorie diet,” “skipped meals/fasted,” “increased exercise,” “diet pills,” “commercial weight loss program,” “gastric surgery/intestinal bypass,” and “other” in the NHS/HPFS. Three more responses (“low fat diet,” “decreased alcohol intake,” and “resumed/increased smoking”) were included in the NHSII questionnaire only. Based on the responses to these questions, participants were categorized into 3 mutually exclusive groups, including those who did not attempt to lose weight, those who lost less than 4.5 kg at a time, and those who lost 4.5+ kg at a time in the past 4 years. We excluded those who reported losing less than 4.5 kg since the WLS information was not collected for these individuals. Participants who did not attempt to lose weight were treated as the reference group. For participants who lost 4.5+ kg intentionally, we first excluded those who lost weight through surgery or other unspecific methods and then excluded NHSII participants who solely used the 3 methods that were not considered in the NHS and HPFS questionnaires to maintain consistency across 3 cohorts. To facilitate analyses and keep interpretation consistent across 3 cohorts, we grouped the WLSs into 7 mutually exclusive categories, including low-calorie diet (LCD), exercise, LCD and exercise, fasting, commercial weight loss program (CWLP), pill, and a combination (two or more) of fasting, CWLP, and pill (FCP for short). The grouping was largely determined by the distribution of individual WLSs as well as the combinations of WLSs in the study population (**S1 Table**).

### Assessment of covariates

In this study, we considered multiple covariates assessed before or in the 1992/1993 questionnaire for multivariate adjustments, including age, ethnicity, height, BMI (weight in kilograms divided by height in meters squared), waist circumference, alcohol intake, smoking status, multivitamin use, physical activity (metabolic equivalents of tasks [METs]), television watching duration, total energy intake, diet quality (Alternative Healthy Eating Index [AHEI] score), family history of diabetes, and history of hypertension or hypercholesterolemia (**S2 Table**).

### Assessment of weight change and type 2 diabetes

Body weight and physician-diagnosed diabetes incidence were collected since baseline and updated biennially. Weight change percentage, defined as [(current weight − baseline weight) / baseline weight] * 100%, was used to measure weight change in the current study. A supplementary questionnaire regarding symptoms, diagnostic tests, and hypoglycemic therapy was mailed to participants who self-reported having physician-diagnosed diabetes to confirm the diagnosis (**S2 Text**).

### Statistical analysis

Data from the 3 cohorts were pooled to maximize statistical power. For the weight change analyses, follow-up was censored when participants reached aged 65 or older on the incidence of diabetes, cardiovascular disease, cancer, death, or pregnancy (NHSII only). For the T2D analyses, person-time for each participant was counted from the return of the 1992/1993 questionnaires to the date of T2D diagnosis, death, last return of a valid follow-up questionnaire, or the end of follow-up (June 2016 for NHS/HPFS; June 2017 for NHSII), whichever came first.

Generalized linear model and generalized estimating equations with unstructured within-subject correlation matrices were used to assess the association of WLSs with baseline body weight and weight change, respectively. Least squares means of body weight and weight change percentages since baseline were calculated to illustrate the trajectory of weight change over time. All available body weights in 1988/1989, 1992/1993, 1994/1995, 1996/1997, and 1998/1999 and the corresponding weight change percentage since baseline were included as a time-varying dependent variable in these models. Because the biennial weight change percentages were mostly differential among WLSs at year 4 and then largely converged to each other at year 10 (1998 for NHS/HPFS or 1999 for NHSII; **S2 Fig**), we focused on weight change by the end of year 4 and year 10, respectively.

Cox proportional hazards model was applied to examine the association of WLSs with the incidence of T2D. The proportional hazards assumption was tested by including the product terms between each exposure indicator and the log-transformed follow-up time. No violation of the assumption was found. Multiple imputation was implemented to minimize the number of missing values in covariates (**S3 Text**). Multiple comparisons were adjusted using Dunnett’s test and false discovery rate when comparing the strength of associations of various WLSs. Given that weight change might be a mediator between WLSs and T2D risks, the extent to which the association might be explained by time-varying BMI was evaluated using a SAS macro %MEDIATE [10].

Stratified analyses were conducted by baseline BMI (<25 kg/m^2^ [lean], 25 to <30 kg/m^2^ [overweight], or ≥30 kg/m^2^ [obese]). Interactions were tested using a likelihood-ratio test comparing models with and without product terms between WLSs and stratifying variables.

We considered several sensitivity analyses. A cubic spline regression model was fitted to delineate the trajectory of hazard ratios (HRs) over follow-up duration. Given the strong impact of ageing on body weight and composition, we repeated the T2D analysis in participants who were <65 years old in 1992 (NHS/HPFS)/1993 (NHSII). To reduce the possibility of reverse-causation, we excluded participants who were diagnosed with T2D in the first 4 years of follow-up (through 1996 [NHS/HPFS]/1997 [NHSII]). To alleviate the concern that the body weight assessments in 1988 (NHS/HPFS) or 1989 (NHSII) may misclassify the long-term weight status before 1988/1989, we redefined individuals who were consistently lean (BMI was less than 25 kg/m^2^ at each biennial follow-up from the initiation of the cohorts to 1988/1989) as the baseline lean group, and the same algorithm was used to define the overweight and obese groups. In another sensitivity analysis, we also used maximum BMI collected before 1992/1993 (1972 to 1992 for HPFS, 1976 to 1992 for NHS, and 1989 to 1993 for NHSII) to define the obesity status. In a sensitivity analysis, we included participants who skipped the WLS question into the reference group. We also stratified the analysis by abdominal obesity (waist circumference ≥102 cm for male and waist circumference ≥88 cm for female). Lastly, in response to peer review comments, we restricted the weight change analyses within participants with complete, valid weight assessments since 1988 (NHS/HPFS)/1989 (NHSII) through 1998 (NHS/HPFS)/1999 (NHSII) to evaluate the impact of missing weight data on associations of interest.

Data were analyzed using SAS software, version 9.4 (SAS Institute). Two-sided multiple comparison adjusted *P* < 0.05 was considered statistically significant.

## Results

Of all participants, including those who lost less than 4.5 kg of body weight, 53.2% (75,201/141,387) reported losing 4.5+ kg intentionally, of whom 13.3% through LCD, 10.7% through exercise, 29.2% through LCD and exercise, 12.6% through fasting, 27.4% through CWLP, 1.9% through pill, and 5.1% through FCP (**S3 Table**). The age-standardized baseline characteristics of the study populations for T2D analyses and weight change analyses are shown in **Tables 1 and S4 and S5**, respectively, and those of participants with/without skipping the WLS question were shown in **S6 Table**.

**Table 1:** Age-standardized characteristics of participants at baseline in the T2D analyses.

Characteristic	Reference	LCD	Exercise	LCD and Exercise	Fasting	CWLP	Pill	FCP
Participants, number	28,979	9,972	8,008	21,971	9,434	20,581	1,433	3,802
Age in 1992 (year)	50.0 (12.3)	51.1 (11.6)	41.8 (9.4)	47.9 (11.2)	45.4 (10.7)	45.9 (10.4)	42.6 (9.5)	42.0 (8.5)
Ethnicity								
White, %	96.6	97.8	96.6	97.5	96.1	97.5	95.4	96.1
African American, %	1.1	1.4	1.5	1.4	2.2	1.6	2.4	2.5
Asian, %	0.6	0.3	0.7	0.4	0.6	0.3	0.9	0.6
Other, %	1.6	0.5	1.0	0.6	1.0	0.6	1.0	0.8
Missing, %	0.1	0	0.1	0.1	0.1	0	0.2	0
BMI (kg/m^2^)	22.5 (3.5)	26.4 (4.9)	24.4 (4.2)	25.5 (4.3)	25.3 (4.7)	27.1 (4.9)	25.8 (4.6)	27.0 (5.3)
Waist circumference (centimeter)	78.1 (11.6)	85.2 (13.5)	80.4 (12.5)	82.7 (12.5)	84.6 (14.1)	84.7 (13.1)	81.9 (13.3)	85.7 (14.4)
Smoking status								
Never smoker, %	58.3	51.8	59.5	56.0	51.5	55.5	55.8	54.0
Past smoker, %	26.2	31.8	27.7	32.4	29.5	32.8	26.7	29.6
Current smoker, %	14.7	15.6	12.4	10.9	18.1	11.4	17.3	16.2
Missing, %	0.8	0.8	0.5	0.7	0.9	0.3	0.1	0.2
Multivitamin use, %	40.3	36.8	44.1	42.6	40.6	41.3	44.9	44.2
Television watching (hour)								
0–1, %	9.2	6.8	10.3	8.0	8.7	6.8	7.8	7.0
2–5, %	29.0	25.8	32.0	28.5	28.2	26.4	30.1	29.3
6–10, %	26.6	26.5	26.4	27.3	26.1	27.2	26.3	26.0
11–20, %	22.8	24.9	19.1	23.2	22.6	24.2	20.9	21.1
21+, %	9.7	12.4	6.4	9.6	9.8	11.5	9.4	10.8
Missing, %	2.7	3.4	5.9	3.5	4.5	3.9	5.6	5.8
Physical activity (METs-hour/week)	11.7 (4.2, 27.7)	8.1 (3.0, 20.2)	19.5 (7.7, 38.8)	15.4 (6.3, 31.2)	13.2 (4.6, 30.4)	10.8 (4.1, 23.6)	12.1 (4.5, 27.8)	12.0 (4.1, 29.0)
AHEI	43.8 (10.7)	45.1 (10.5)	46.7 (10.6)	47.0 (10.5)	43.9 (10.4)	47.3 (10.6)	43.8 (10.1)	44.9 (10.6)
Total energy intake (kilocalorie/day)	1,878 (562)	1,794 (558)	1,810 (558)	1,794 (539)	1,800 (593)	1,765 (530)	1,725 (548)	1,761 (566)
Alcohol consumption (gram/day)	1.8 (0, 7.6)	1.5 (0, 6.4)	1.1 (0, 4.9)	1.8 (0, 6.3)	1.9 (0, 7.3)	1.1 (0, 4.0)	1.1 (0, 4.7)	1.1 (0, 4.7)
Self-reported hypertension, %	13.5	23.9	11.1	19.8	17.6	20.8	14.2	17.7
Self-reported hypercholesterolemia, %	24.2	33.7	21.5	30.6	26.8	31.7	27.2	31.3
Family history of diabetes, %	18.6	23.6	17.8	22.5	21.0	23.4	22.2	22.9

Values are means (standard deviation) or medians (Q25, Q75) for continuous variables; percentages for categorical variables, and are standardized to the age distribution of the study population. Values of polytomous variables may not sum to 100% due to rounding.

AHEI, Alternative Healthy Eating Index; BMI, body mass index; CWLP, commercial weight loss program; FCP, select at least 2 strategies among fasting, CWLP, and pill; kg/m^2^, kilogram per square meter; LCD, low-calorie diet; MET, metabolic equivalent of tasks; T2D, type 2 diabetes.

### Weight loss strategies and weight change

**Fig 1:**
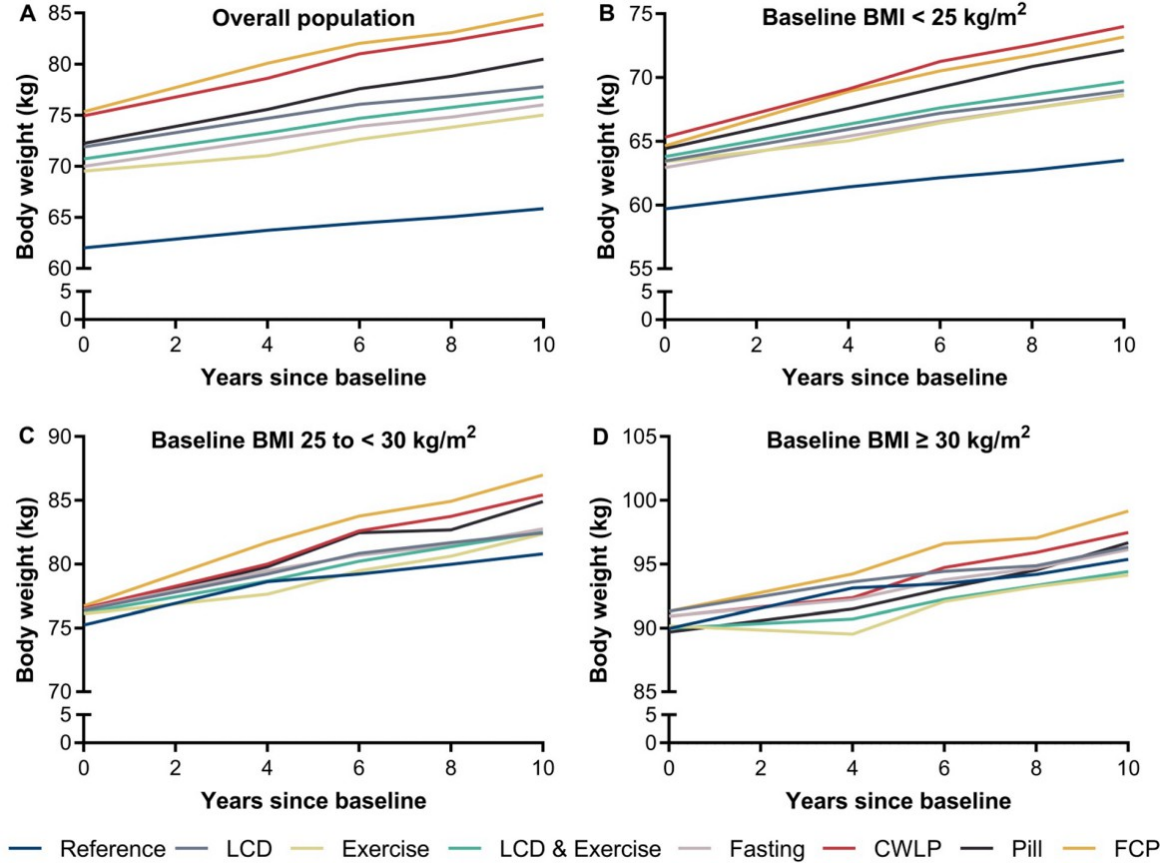
WLSs and weight change trajectories by baseline BMI. (**A**) Overall population. (**B**) BMI <25 kg/m^2^. (**C**) BMI 25 to <30 kg/m^2^. (**D**) BMI ≥30 kg/m^2^. All body weights were calculated based on baseline weight and weight change percentage since baseline. For weight change percentage, the multivariable model was adjusted for cohort (HPFS, NHS, or NHSII), age (in month, continuous), ethnicity (white, African American, Asian, or other), baseline body weight (in kilogram, continuous), baseline waist circumference (in centimeter, continuous), physical activity (in quintiles), television watching (0–1, 2–5, 6–10, 11–20, or >20 hour/week), smoking status (never, past, or current smokers), alcohol intake (0, <5.0, 5.0–9.9, 10.0–14.9, 15.0–29.9, or >30.0 gram/day), hypertension (yes or no), hypercholesterolemia (yes or no), family history of diabetes (yes or no), multivitamin use (yes or no), AHEI score (in quintiles), and total energy intake (in quintiles) before weight loss. For baseline body weight, all abovementioned covariates were adjusted for except that body weight and waist circumference were replaced with height (in meter, continuous). AHEI, Alternative Healthy Eating Index; BMI, body mass index; CWLP, commercial weight loss program; FCP, select at least 2 strategies among fasting, CWLP, and pill; HPFS, Health Professionals Follow-Up Study; kg, kilogram; kg/m^2^, kilogram per square meter; LCD, low-calorie diet; NHS, Nurses’ Health Study; WLS, weight loss strategy. 1 kg = 2.2 lbs.

The temporal trend of body weight according to WLSs is shown in **Fig 1A**. Regardless of whether or not participants tried to lose weight or what WLSs they had adopted, their body weight, on average, increased over time. However, the weight gain trajectories were differential among WLS groups. By 10 years of follow-up, all WLS groups were associated with more weight gain than the reference group (ranging from 1.7% for exercise to 6.6% for FCP) (**Tables 2 and S7**).

**Table 2:** Baseline weight and weight change percentage since baseline across different WLSs.

WLS	No. of Participants	Adjusted for Age						Adjusted for Multiple Variables					
		Difference of Baseline Weight (kg)	*P* value	Difference of Weight Change Percentage (%)				Difference of Baseline Weight (kg)	*P* value	Difference of Weight Change Percentage (%)			
				Four Years since Baseline	*P* value	Ten Years since Baseline	*P* value			Four Years since Baseline	*P* value	Ten Years since Baseline	*P* value
**Overall Population**													
Reference	24,164	0 (Reference)	-	0 (Reference)	-	0 (Reference)	-	0 (Reference)	-	0 (Reference)	-	0 (Reference)	-
LCD	8,326	10.6 (10.2, 11.0)	<0.001	1.0 (0.8, 1.2)	<0.001	1.9 (1.6, 2.2)	<0.001	9.9 (9.6, 10.2)	<0.001	1.1 (0.9, 1.3)	<0.001	2.0 (1.7, 2.4)	<0.001
Exercise	7,658	7.3 (7.0, 7.6)	<0.001	−0.7 (−0.9, −0.5)	<0.001	1.6 (1.3, 1.9)	<0.001	7.5 (7.2, 7.8)	<0.001	−0.6 (−0.9, −0.4)	<0.001	1.7 (1.4, 2.0)	<0.001
LCD and Exercise	19,773	8.4 (8.2, 8.6)	<0.001	0.7 (0.5, 0.8)	<0.001	2.3 (2.1, 2.5)	<0.001	8.7 (8.5, 8.9)	<0.001	0.7 (0.6, 0.9)	<0.001	2.4 (2.2, 2.6)	<0.001
Fasting	8,796	9.3 (9.0, 9.7)	<0.001	0.7 (0.5, 0.9)	<0.001	2.2 (1.9, 2.5)	<0.001	8.0 (7.7, 8.3)	<0.001	0.9 (0.7, 1.1)	<0.001	2.4 (2.1, 2.7)	<0.001
CWLP	19,269	12.1 (11.8, 12.3)	<0.001	2.1 (1.9, 2.3)	<0.001	5.8 (5.5, 6.0)	<0.001	12.9 (12.7, 13.1)	<0.001	2.1 (1.9, 2.3)	<0.001	5.7 (5.4, 6.0)	<0.001
Pill	1,388	9.1 (8.4, 9.9)	<0.001	2.0 (1.5, 2.5)	<0.001	5.2 (4.4, 5.9)	<0.001	10.2 (9.6, 10.9)	<0.001	1.8 (1.2, 2.4)	<0.001	5.2 (4.4, 6.0)	<0.001
FCP	3,736	13.1 (12.6, 13.6)	<0.001	3.6 (3.2, 4.0)	<0.001	6.8 (6.2, 7.3)	<0.001	13.3 (12.9, 13.8)	<0.001	3.5 (3.1, 3.9)	<0.001	6.6 (6.0, 7.1)	<0.001
**Baseline BMI <25 kg/m** ^**2**^													
Reference	20,077	0 (Reference)	-	0 (Reference)	-	0 (Reference)	-	0 (Reference)	-	0 (Reference)	-	0 (Reference)	-
LCD	3,915	3.2 (3.0, 3.5)	<0.001	1.4 (1.1, 1.6)	<0.001	2.6 (2.3, 3.0)	<0.001	3.8 (3.6, 3.9)	<0.001	1.0 (0.7, 1.3)	<0.001	2.3 (1.9, 2.7)	<0.001
Exercise	5,060	3.6 (3.4, 3.9)	<0.001	−0.2 (−0.4, 0.1)	0.64	2.0 (1.7, 2.3)	<0.001	3.7 (3.6, 3.8)	<0.001	−0.4 (−0.6, −0.1)	0.02	1.8 (1.5, 2.2)	<0.001
LCD and Exercise	10,780	3.5 (3.3, 3.7)	<0.001	1.4 (1.2, 1.6)	<0.001	3.1 (2.9, 3.4)	<0.001	4.1 (4.0, 4.2)	<0.001	1.1 (0.9, 1.3)	<0.001	2.8 (2.6, 3.1)	<0.001
Fasting	5,058	3.3 (3.0, 3.5)	<0.001	1.2 (1.0, 1.5)	<0.001	2.9 (2.6, 3.3)	<0.001	3.2 (3.1, 3.4)	<0.001	1.0 (0.7, 1.2)	<0.001	2.7 (2.4, 3.1)	<0.001
CWLP	7,867	4.4 (4.2, 4.6)	<0.001	3.5 (3.3, 3.7)	<0.001	7.7 (7.4, 8.0)	<0.001	5.6 (5.5, 5.7)	<0.001	2.9 (2.6, 3.1)	<0.001	7.0 (6.6, 7.3)	<0.001
Pill	731	3.7 (3.2, 4.2)	<0.001	2.4 (1.8, 3.0)	<0.001	6.0 (5.0, 7.0)	<0.001	4.7 (4.4, 5.0)	<0.001	2.0 (1.3, 2.6)	<0.001	5.6 (4.6, 6.6)	<0.001
FCP	1,576	4.2 (3.8, 4.5)	<0.001	4.4 (3.9, 5.0)	<0.001	7.6 (6.8, 8.3)	<0.001	5.0 (4.7, 5.2)	<0.001	3.7 (3.1, 4.3)	<0.001	6.9 (6.1, 7.6)	<0.001
**Baseline BMI 25 to <30 kg/m** ^**2**^													
Reference	3,196	0 (Reference)	-	0 (Reference)	-	0 (Reference)	-	0 (Reference)	-	0 (Reference)	-	0 (Reference)	-
LCD	2,783	0.6 (0.1, 1.1)	0.09	−0.5 (−0.9, −0.1)	0.09	0.8 (0.1, 1.4)	0.11	1.2 (1.0, 1.4)	<0.001	−0.9 (−1.2, −0.5)	<0.001	0.4 (−0.2, 1.1)	0.82
Exercise	1,857	0.4 (−0.2, 0.9)	0.65	−2.1 (−2.6, −1.6)	<0.001	1.0 (0.3, 1.7)	0.02	0.9 (0.7, 1.1)	<0.001	−2.5 (−3.0, −2.1)	<0.001	0.7 (0.0, 1.5)	0.23
LCD and Exercise	6,392	0.0 (−0.4, 0.4)	1.0	−0.9 (−1.2, −0.6)	<0.001	1.2 (0.7, 1.7)	<0.001	0.9 (0.8, 1.1)	<0.001	−1.2 (−1.6, −0.9)	<0.001	0.9 (0.4, 1.4)	0.004
Fasting	2,565	2.6 (2.1, 3.1)	<0.001	−0.6 (−1.0, −0.1)	0.07	0.9 (0.2, 1.5)	0.05	1.2 (1.0, 1.4)	<0.001	−0.6 (−1.0, −0.1)	0.07	0.9 (0.2, 1.5)	0.05
CWLP	7,049	−1.3 (−1.7, −0.9)	<0.001	0.7 (0.4, 1.1)	<0.001	4.8 (4.3, 5.3)	<0.001	1.3 (1.1, 1.5)	<0.001	0.0 (−0.4, 0.4)	1.0	4.2 (3.6, 4.7)	<0.001
Pill	434	−2.2 (−3.1, −1.4)	<0.001	1.3 (0.3, 2.3)	0.08	4.3 (2.9, 5.7)	<0.001	1.1 (0.7, 1.5)	<0.001	0.0 (−1.1, 1.2)	0.98	3.8 (2.3, 5.2)	<0.001
FCP	1,265	−0.7 (−1.3, −0.1)	0.13	2.8 (2.1, 3.5)	<0.001	7.0 (6.0, 8.0)	<0.001	1.5 (1.2, 1.7)	<0.001	2.0 (1.3, 2.7)	<0.001	6.0 (5.0, 7.0)	<0.001
**Baseline BMI ≥30 kg/m** ^**2**^													
Reference	891	0 (Reference)	-	0 (Reference)	-	0 (Reference)	-	0 (Reference)	-	0 (Reference)	-	0 (Reference)	-
LCD	1,628	1.6 (0.5, 2.7)	0.03	−0.9 (−1.6, −0.3)	0.03	−0.5 (−1.6, 0.7)	0.94	1.3 (0.5, 2.2)	0.01	−1.0 (−1.7, −0.3)	0.02	−0.5 (−1.6, 0.7)	0.90
Exercise	741	−0.4 (−1.8, 1.0)	0.98	−4.1 (−5.0, −3.1)	<0.001	−1.6 (−3.0, −0.2)	0.11	0.2 (−0.8, 1.2)	>0.99	−4.2 (−5.1, −3.2)	<0.001	−1.5 (−2.9, −0.1)	0.12
LCD and Exercise	2,601	−0.6 (−1.6, 0.5)	0.80	−2.6 (−3.3, −2.0)	<0.001	−1.2 (−2.2, −0.2)	0.12	0.0 (−0.7, 0.8)	1.0	−2.7 (−3.3, −2.0)	<0.001	−1.0 (−2.1, 0.0)	0.16
Fasting	1,173	2.0 (0.8, 3.2)	0.005	−2.3 (−3.0, −1.5)	<0.001	−0.6 (−1.8, 0.6)	0.90	1.0 (0.1, 1.9)	0.15	−2.1 (−2.8, −1.3)	<0.001	−0.3 (−1.5, 0.9)	>0.99
CWLP	4,353	0.0 (−1.0, 1.0)	1.0	−1.8 (−2.3, −1.2)	<0.001	1.3 (0.4, 2.3)	0.04	1.0 (0.2, 1.7)	0.07	−1.9 (−2.5, −1.3)	<0.001	1.3 (0.3, 2.3)	0.06
Pill	223	−1.7 (−3.6, 0.3)	0.39	−1.2 (−2.7, 0.3)	0.51	2.0 (−0.2, 4.2)	0.30	−0.3 (−1.8, 1.2)	>0.99	−1.5 (−3.0, 0.1)	0.32	1.8 (−0.4, 4.0)	0.37
FCP	895	0.7 (−0.6, 2.1)	0.77	0.1 (−0.8, 1.0)	1.0	2.8 (1.4, 4.2)	<0.001	1.3 (0.3, 2.3)	0.05	−0.3 (−1.2, 0.7)	>0.99	2.6 (1.2, 4.0)	0.002

For weight change percentage, the multivariable model was adjusted for cohort (HPFS, NHS, or NHSII), age (in month, continuous), ethnicity (white, African American, Asian, or other), baseline body weight (in kg, continuous), baseline waist circumference (in centimeter, continuous), physical activity (in quintiles), television watching (0–1, 2–5, 6–10, 11–20, or > 20 hour/week), smoking status (never, past, or current smokers), alcohol intake (0, <5.0, 5.0–9.9, 10.0–14.9, 15.0–29.9, or >30.0 gram/day), hypertension (yes or no), hypercholesterolemia (yes or no), family history of diabetes (yes or no), multivitamin use (yes or no), AHEI score (in quintiles), and total energy intake (in quintiles) before weight loss. For baseline body weight, all abovementioned covariates were adjusted for except that body weight and waist circumference were replaced with height (in meter, continuous). *P* for interaction for overall and individual WLSs were less than 0.001. *P* values were adjusted using Dunnett’s test.

AHEI, Alternative Healthy Eating Index; BMI, body mass index; CWLP, commercial weight loss program; FCP, select at least 2 strategies among fasting, CWLP, and pill; HPFS, Health Professionals Follow-Up Study; kg, kilogram; kg/m^2^, kilogram per square meter; LCD, low-calorie diet; NHS, Nurses’ Health Study; No., number; WLS; weight loss strategy.

1 kg = 2.2 lbs.

### Weight loss strategies and type 2 diabetes

**Fig 2:**
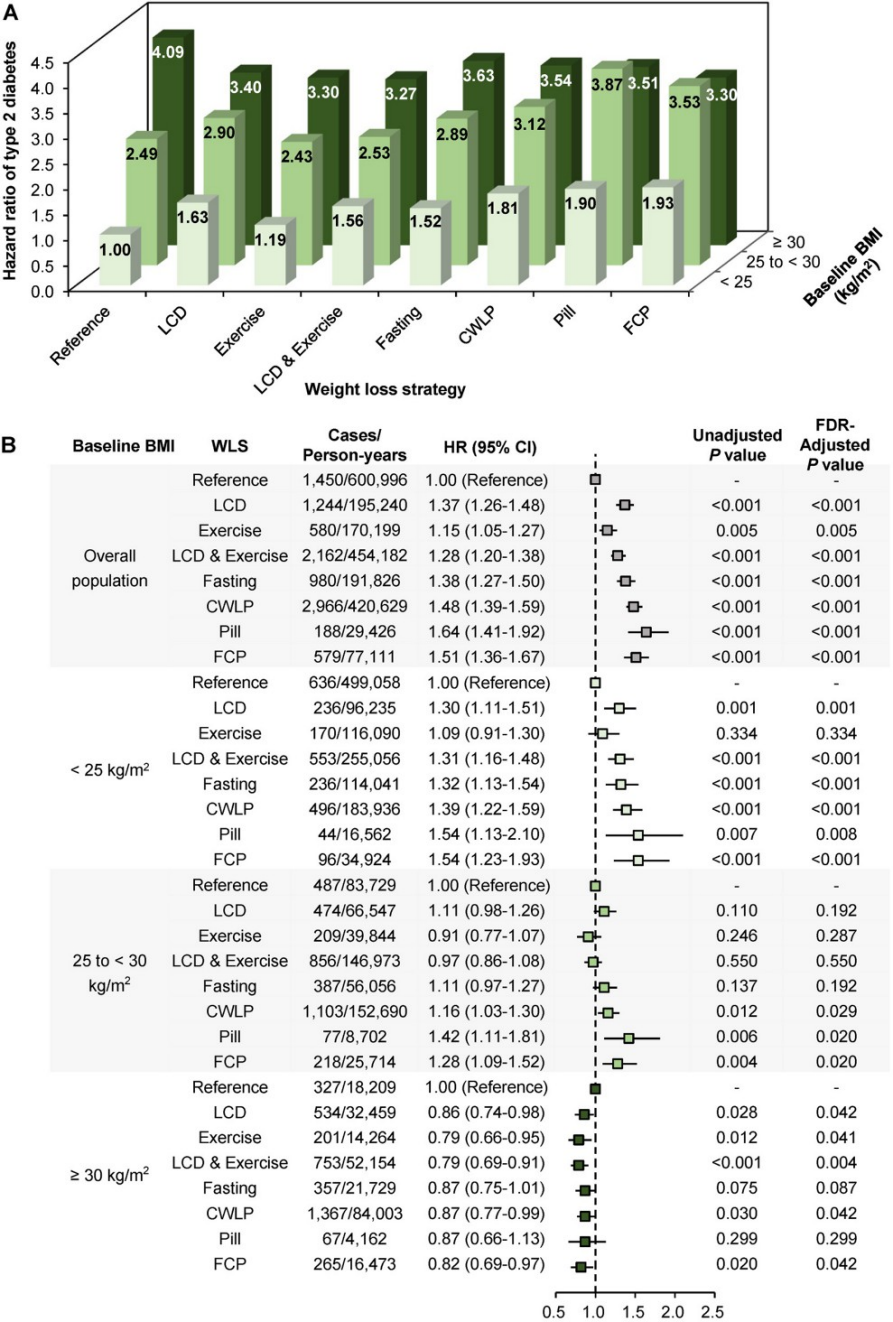
Pooled HRs for association between WLSs and the incidence of T2D by baseline BMI. (**A**) All BMI categories have the same reference (individuals whose BMI was <25 kg/m^2^ and who did not attempt to lose weight). (**B**) Each BMI category has its own reference. Multivariable models were adjusted for cohort (HPFS, NHS, or NHSII), age (in month, continuous), ethnicity (white, African American, Asian, or other), baseline BMI (in kg/m^2^, continuous), baseline waist circumference (in centimeter, continuous), physical activity (in quintiles), television watching (0–1, 2–5, 6–10, 11–20, or >20 hour/week), smoking status (never, past, or current smokers), alcohol intake (0, <5.0, 5.0–9.9, 10.0–14.9, 15.0–29.9, or >30.0 gram/day), hypertension (yes or no), hypercholesterolemia (yes or no), family history of diabetes (yes or no), multivitamin use (yes or no), AHEI score (in quintiles), and total energy intake (in quintiles) before weight loss. *P* for interaction for overall and individual WLSs were less than 0.01. AHEI, Alternative Healthy Eating Index; BMI, body mass index; CI, confidence interval; CWLP, commercial weight loss program; FCP, select at least 2 strategies among fasting, CWLP, and pill; FDR, false discovery rate; HPFS, Health Professionals Follow-Up Study; HR, hazard ratio; kg/m^2^, kilogram per square meter; LCD, low-calorie diet; NHS, Nurses’ Health Study; T2D, type 2 diabetes; WLS, weight loss strategy.

During 2.14 million person-years of follow-up, 10,149 incident cases of T2D were observed (**Fig 2**). After multivariate adjustments, all WLSs were significantly associated with a higher risk of developing T2D. In comparison with the reference group, the HR varied from 1.15 (95% confidence interval [CI] 1.05, 1.27; *P* = 0.005) for exercise to 1.64 (95% CI 1.41, 1.92; *P* < 0.001) for pill. The proportions of the association between WLSs and T2D risk mediated by time-varying BMI after weight loss ranged from 15.6% (95% CI 7.7%, 29.0%; *P* < 0.001) for exercise to 46.8% (95% CI 37.7%, 56.1%; *P* < 0.001) for FCP (**S8 Table**).

### Modification by baseline body mass index

Among participants who lost 4.5+ kg, those with higher baseline BMI were less likely to choose exercise, LCD and exercise, or fasting and more likely to choose LCD, CWLP, and FCP than leaner participants (**S3 Table**). We observed significant interactions between overall/individual WLSs and baseline BMI on the associations of interest (*P*_interaction_ < 0.001). Among individuals with obesity, all WLSs tended to be associated with less weight gain during the first 4 years of follow-up, whereas among lean individuals, all WLSs except exercise tended to be associated with more weight gain than the reference group (**Table 2**). Of all WLSs, exercise was associated with the least absolute weight change percentage in all BMI categories: −0.7%, 2.0%, and 2.6% among individuals who were originally obese, overweight, and lean, respectively (**S3 Fig**). During the extended follow-up (10 years), CWLP, pill, and FCP had more weight gain than the other WLSs for all BMI categories. The weight change over time is shown in **Fig 1B–1D**.

Similar effect modification by baseline BMI on the associations between WLSs and T2D was also observed (*P*_interaction_ < 0.01). Compared with the reference group, all WLSs tended to be associated with a lower risk of developing T2D in individuals with obesity (HRs ranging from 0.79 to 0.87), whereas in lean individuals, the opposite pattern of association was observed (HRs ranging from 1.09 to 1.54) (**Fig 2)**. Of all WLSs, exercise was the only WLS that was not significantly associated with T2D risk among lean participants (HR 1.09 [95% CI 0.91, 1.30; *P* = 0.33]). The effect modification remained when BMI was treated as a continuous variable. The T2D risk compared with the reference group was significantly lowered by 4.0% (95% CI 2.6%, 5.4%; *P* < 0.001) for exercise to 8.1% (6.8%, 9.5%; *P* < 0.001) for FCP for each unit increment of baseline BMI (**S9 Table**).

### Secondary and sensitivity analyses

HRs for T2D across different WLSs were generally consistent over time (**S4 Fig**). Results were similar when aged participants or those who were diagnosed T2D in the first 4-year follow-up were excluded from the analyses, when the analysis was based on individuals who were consistently lean/overweight/obese before 1988/1989, when the stratification was based on maximum BMI before 1992/1993, or when participants who skipped the WLS question were included into the reference group (**S5–S9 Figs** and **S10–S12 Tables**). When analyses were stratified by baseline abdominal obesity status, by 10 years of follow-up, all WLSs tended to associate with more weight gain, and the association was stronger in individuals without abdominal obesity (*P*_interaction_ < 0.001; **S13 Table** and **S10 and S11 Figs**). Accordingly, all WLSs tended to associate with a higher risk of T2D in participants without abdominal obesity, with HRs varying from 1.02 to 1.69 (**S12 Fig**). Among individuals with abdominal obesity, none of the WLSs was associated with the risk of T2D. The T2D risk compared with the reference group significantly reduced by 1.4% (95% CI 0.8% to 2.1%; *P* < 0.001) for exercise to 2.9% (95% CI 2.2% to 3.6%; *P* < 0.001) for FCP for each unit increment of waist circumference (**S14 Table**). Lastly, the results of weight changes among participants with complete weight assessments (**S15 Table**) remained largely unchanged, compared with those in **Table 2**.

## Discussion

### Principal findings

In 3 cohorts of US men and women, about half of participants reported intentionally losing 4.5+ kg of body weight using various strategies, ranging from lifestyle modifications (e.g., LCD, exercise, and their combinations) to fasting or other commercial interventions (e.g., CWLP and pill). The primary finding is that the associations of various WLSs with weight change and T2D risk are dependent on body weight status before weight loss. Specifically, among individuals with obesity, compared with those who did not attempt to lose weight, those who lost 4.5+ kg gained less weight and had lower T2D risk, regardless of the WLSs used to achieve the weight loss. This pattern of favorable associations was less clear among overweight individuals and even reversed among lean individuals. Of all WLSs, exercise was associated with the least weight gain and the lowest T2D risk among individuals with obesity.

### Comparison with other studies

Weight maintenance after weight loss is notoriously challenging [5]. In our cohorts, we observed universal weight gain from baseline across all groups that underwent weight loss, although different WLSs were associated with differential weight gain trajectories. This is in accordance with previous findings in a systematic review of prospective studies with a minimum 3-year follow-up after weight loss. This review indicated that most individuals who had lost at least 5% of body weight using diet, diet and exercise, or cognitive behavioral treatment regained weight to the preintervention baseline weight without sustained intervention after approximately 4 years [6]. Nonetheless, of all strategies, exercise appeared to associate with the least weight gain in our study. This was supported by the result of a trial where the exercise group lost 2.9 kg after 1-year intervention and regained 0.2 kg after another year without intervention, whereas the diet group lost 6.8 kg but regained 7.7 kg, and the diet and exercise group lost 8.9 kg and regained 6.7 kg [11]. It is worth noting that, in addition to the inclusion of a reference group and the use of prospective study design, our study is substantially different from these prior studies in that our focus was long-term weight change since baseline, which is not necessarily equivalent to weight maintenance (weight change since the end of weight loss) in intervention study settings. Nonetheless, the current evidence thus far collectively highlights the role of exercise in long-term weight control after intentional weight loss [12].

Although all strategies except exercise were associated with more weight gain, we observed a gradient in the weight gain trajectory in that participants who took pills, used CWLPs or their combinations with fasting (FCP) tended to gain more weight than those who followed a LCD or fasted. Evidence for comparisons of long-term weight changes between CWLPs or pills and other WLSs is scarce. Nonetheless, this observation is in line with previous studies showing that individuals who used a self-guided approach were better at maintaining their initial weight loss compared with those who commenced a CWLP [13]. Regarding LCD versus fasting as weight loss methods, a pilot study suggested that there was no significant difference in weight regain between intermittent fasting and daily caloric restriction, but more % fat mass was lost and more % lean mass was regained by the end of the 24-week follow-up in the fasting group [14]. In contrast, a recent randomized trial showed that time-restricting eating did not lead to additional weight loss than calorie restriction alone within 12 months [15]. Apparently, more research is needed to further compare the efficacy of different WLSs on promoting weight loss.

Intriguingly, the pattern of associations with T2D risk clearly mirrors that for long-term weight change in that exercise was associated with the least-elevated T2D risk. We further estimated that a significant proportion of these positive associations might be ascribed to the weight changes following weight loss attempts, highlighting the role of long-term weight control following weight loss in the primary prevention of T2D.

Although it is not entirely clear why exercise may outperform other WLSs, some potential mechanisms may explain the less weight gain and more favorable T2D risk associated with exercise. A series of compensatory physiological adaptations favoring weight regain are triggered by weight loss, such as increases in orexigenic hormones (e.g., ghrelin) and fat accumulation and decreases in anorexigenic hormones (e.g., leptin, cholecystokinin, peptide YY) and energy expenditure [16–19]. Exercise was demonstrated to mitigate weight regain via counteracting some of these adaptations. For example, exercise has been reported to restore the hormone perturbations, increase energy expenditure and fat oxidation, and reduce the adipocyte size, which has not been observed in the context of caloric restriction [19–21]. In addition, exercise was suggested to facilitate weight maintenance by breaking the vicious cycle of stress and obesity [22]. Importantly, exercise might be more sustainable. In a weight loss trial, 44% of participants in the exercise group reported exercising often after intervention, but only 6.7% in the diet group reported adhering often to previous dietary recommendations [11]. The better retention of fat-free mass (FFM) and greater fat reduction compared to caloric restriction may explain the minimally increased T2D risk associated with exercise, given the potential protective effect of FFM and the adverse effect of excess body fat on T2D [23,24]. A systematic review summarized that exercise was shown to decrease the percentage weight loss as FFM (%FFML), whereas the degree of caloric restriction was positively associated with %FFML [25]. Another systematic review revealed that 5% loss in body weight was associated with 21.3% reduction in visceral adiposity after exercise but with 13.4% reduction after a hypocaloric diet, and exercise was related to 6.1% decrease in visceral adiposity even in the absence of weight loss while the corresponding number was only 1.1% for a hypocaloric diet [26]. Moreover, exercise has been shown to improve insulin sensitivity independent of weight loss [27].

The effectiveness of CWLP and pills on weight maintenance particularly depends on the duration of use and degree of compliance [28,29]. However, in free-living participants, the retention rate decreased dramatically over time: 73% at 1 month, 42% at 3 months, 22% at 6 months, and 6.6% at 12 months in the Jenny Craig Platinum program [30]. The greater weight regain in the CWLP group compared with conventional self-directed WLSs might partially be ascribed to their differences regarding to confidence, motivation, and cost [13], which may result in less sustainable low-calorie dietary habits after weight loss [16]. In the early 1990s, the most popular diet pills, such as phentermine, fenfluramine, diethylpropion, and others, were restricted to short-term use (a few weeks) because of safety concerns [31,32], such as addiction and side effects of elevation of heart rate and dizziness [33]. Weight regain is common once the medication is terminated unless the medication is combined with healthy eating habits and increasing physical activity [34]. Current long-term diet pills (e.g., orlistat, top-dose [15/92 mg] phentermine plus topiramate-extended release), when used in adjunction with lifestyle interventions, have been indicated to increase the likelihood of achieving clinically meaningful 1-year weight loss compared with placebo [33]. As for fasting, adherence to various regimens was inconclusive [35]. Some investigators speculated that intermittent fasting might reduce adaptive responses induced by energy restriction by regularly raising energy intake on fed days, but evidence was limited with insufficient power [36].

Despite the differences among WLSs used to achieve weight loss, we observed universal health benefits associated with all WLSs among individuals with obesity. Another noteworthy point is that among individuals with obesity, although the body weight of LCD, fasting, CWLP, and FCP groups was consistently larger than that of the reference group after weight loss (**Fig 1D**), their T2D risk continued to be lower, suggesting that 4.5+ kg of weight loss, even though transient, can still lead to a decreased T2D risk in individuals with obesity in a long run. This notion was also supported by results from the Diabetes Prevention Program, which suggested that even a one-time weight loss intervention could have lasting effects on reducing T2D risk for at least 10 years [37]. This is also the case for overweight individuals who lost 4.5+ kg through exercise or LCD and exercise. Similar long-lasting beneficial effects of limited duration lifestyle interventions on T2D incidence were demonstrated in several well-known trials focusing on T2D prevention among those with overweight/obesity [38].

Weight loss attempts are remarkably prevalent among lean individuals [4], as was observed in our study population (50.7% [34,987/68,946] of participants who lost 4.5+ kg intentionally were lean at baseline), which might be partly attributed to their weight misperception influenced by the sociocultural pressure of being lean [39]. Indeed, as a previous study reported, 53.8% of lean women and 22.7% of lean men perceived themselves as being slightly overweight, and 58.1% and 24.7% of them tried to lose weight, respectively [40]. Our observations of divergent pattern of associations by baseline BMI status were consistent with findings from Finnish cohorts that the risk of having a major weight gain (>10 kg) or increases in BMI or waist circumference in lean dieters versus lean non-dieters was generally higher than that in overweight counterparts [7,41]. Other prospective studies also demonstrated stronger inverse associations between exercise and T2D risk in overweight participants than their lean counterparts [8,9]. For example, the relative risks of diabetes in the exercise group compared with the sedentary group were 1.22, 0.87, 0.69, and 0.61 from the lowest to the highest quartiles of BMI [9]. As a result, the high proportion of lean individuals in those who lost 4.5+ kg in our population, together with the effect modifications by BMI, may explain our unintuitive finding that participants who lost 4.5+ kg were likely to gain more weight and have higher risk of T2D than those who did not attempt to lose weight. The reason that might explain the modification of baseline BMI was that fat overshooting and FFM loss were more severe in lean individuals than in those with overweight or obesity upon weight loss [23,24,42]. A critical mechanism could be that in the process of weight regain, fat is fully regained much earlier than FFM, and such desynchronization results in a state of hyperphagia that persists until FFM is fully recovered, during which fat continues to accumulate, leading to fat (and weight) overshooting [42]. A reanalysis of data from the Minnesota Semi-Starvation Experiment further showed that the extent of fat overshooting was inversely correlated with the initial percentage of body fat [42]. Consistently, hyperphagic responses followed by long-term exercise have been reported in lean individuals but not in overweight individuals or those with obesity [43,44]. In addition, the %FFML usually exceeded 35% in normal-weight individuals, while the number was approximately 20% to 30% in the overweight/obese [45]. Taken together, the current and prior evidence suggests that lean individuals may not benefit from intentional weight loss, possibly due to the physiological process that predisposes lean individuals to fat overshooting or excess weight regain after they lose weight.

### Strengths and limitations

To our knowledge, this is the first study that comprehensively examined the long-term associations of multiple WLSs with weight change and T2D risk in a large group of free-living individuals in a real-world setting. Another noteworthy strength is that we only considered intentional weight loss through the strategies and thus minimized the strong impact of chronic diseases and other causes of unintentional weight loss on associations of interest. Several limitations are worth mentioning. First, we could not further distinguish the methods in each broad category of WLSs, which can be rather heterogeneous. As such, what we observed are “average” associations that may not be fully generalizable to a more specific strategy. Second, the homogeneous ethnicity and socioeconomic status, although can help alleviate the confounding by these factors, further limit the generalizability of our observations to other populations with different characteristics. Third, we were unable to evaluate the impact of previous or subsequent WLSs on the associations of interest. Fourth, we did not assess the exact amount nor the exact time of weight loss. We thus cannot assess the role of weight loss amount on weight gain and T2D risk and may pool person-time of heterogeneous scenarios (e.g., weight change right after weight loss versus weight change after several years since weight loss). Lastly, as for any epidemiological studies, we cannot exclude the role of residual/unmeasured confounding or chance in our observations. More studies are needed to further elucidate these important associations.

## Conclusion and policy implications

In conclusion, in individuals with obesity, losing 4.5+ kg of body weight intentionally was associated with less weight gain and lower T2D risk, regardless of the methods used to achieve the weight loss. However, for individuals who were lean, losing 4.5+ kg was not associated with these health benefits. Of all WLSs, exercise was optimal for long-term weight control and T2D prevention. Our data support current guidelines for body weight management, such as that issued by the Obesity Society, which recommend a weight loss of 5% to 10% of baseline weight for individuals who are overweight or obese and exercise of 200 to 300 minutes per week to maintain the weight loss [46].

## Supporting information

S1 Checklist STROBE statement. (DOCX)

S1 Text Study protocol. (DOCX)

S2 Text The confirmation of self-reported type 2 diabetes. (DOCX)

S3 Text Multiple imputation of the covariates. (DOCX)

S1 Table Components of the weight loss strategies in the type 2 diabetes analyses. (DOCX)

S2 Table Data source of the covariates. (DOCX)

S3 Table Age-standardized proportions of weight loss strategies by baseline body mass index in the type 2 diabetes analyses. (DOCX)

S4 Table Age-standardized characteristics of participants before weight loss in the type 2 diabetes analyses. (DOCX)

S5 Table Age-standardized characteristics of participants before weight loss in the weight change analyses. (DOCX)

S6 Table Comparison of characteristics before weight loss between participants who skipped the weight loss strategy question and those who did not. (DOCX)

S7 Table Baseline weight and weight change percentage since baseline across different weight loss strategies (*P* values were unadjusted). (DOCX)

S8 Table Proportions (95% CIs) of the association between weight loss strategies and type 2 diabetes mediated by time-varying body mass index. (DOCX)

S9 Table The hazard ratio of type 2 diabetes reduced by each unit increment of baseline body mass index. (DOCX)

S10 Table Weight loss strategies and weight change percentages (consistently lean/overweight/obese before 1988/1989). (DOCX)

S11 Table Weight loss strategies and weight change percentages (maximum body mass index before 1992/1993). (DOCX)

S12 Table Weight loss strategies and weight change percentages (participants who skipped the weight loss strategy question were included into the reference group). (DOCX)

S13 Table Baseline weight and weight change percentage since baseline across different weight loss strategies stratified by baseline abdominal obesity status. (DOCX)

S14 Table The hazard ratio of type 2 diabetes reduced by each unit increment of baseline waist circumference. (DOCX)

S15 Table Baseline weight and weight change percentage since baseline across different weight loss strategies (complete case analysis). (DOCX)

S1 Fig Flow chart of participants. (PDF)

S2 Fig Weight change trajectories of different weight loss strategies. (PDF)

S3 Fig Weight loss strategies and absolute weight change percentages since baseline stratified by baseline body mass index. (PDF)

S4 Fig Pooled hazard ratios of type 2 diabetes by time according to weight loss strategies. (PDF)

S5 Fig Pooled hazard ratios for association between weight loss strategies and the incidence of type 2 diabetes in participants <65 years old. (PDF)

S6 Fig Pooled hazard ratios for association between weight loss strategies and the incidence of type 2 diabetes (participants who were diagnosed type 2 diabetes in the first 4-year follow-up were excluded from the analysis). (PDF)

S7 Fig Pooled hazard ratios for association between weight loss strategies and the incidence of type 2 diabetes in participants who were consistently lean/overweight/obese before 1988/1989. (PDF)

S8 Fig Pooled hazard ratios for association between weight loss strategies and the incidence of type 2 diabetes stratified by maximum BMI before 1992/1993. (PDF)

S9 Fig Pooled hazard ratios for association between weight loss strategies and the incidence of type 2 diabetes (participants who skipped the weight loss strategy question were included into the reference group). (PDF)

S10 Fig Weight loss strategies and weight change trajectories by baseline abdominal obesity status. (PDF)

S11 Fig Weight loss strategies and absolute weight change percentages since baseline stratified by baseline abdominal obesity status. (PDF)

S12 Fig Pooled hazard ratios for association between weight loss strategies and the incidence of type 2 diabetes stratified by baseline abdominal obesity status. (PDF)

## Abbreviations

AHEI: Alternative Healthy Eating Index
BMI: body mass index
CI: confidence interval
CWLP: commercial weight loss program
FCP: fasting, commercial weight loss program, or pill
FFM: fat-free mass
%FFML: percentage weight loss as fat-free mass
HPFS: Health Professionals Follow-Up Study
HR: hazard ratio
LCD: low-calorie diet
MET: metabolic equivalent of tasks
NHS: Nurses’ Health Study
T2D: type 2 diabetes
WLS: weight loss strategy

## References

[1] Hales CM, Carroll MD, Fryar CD, Ogden CL. Prevalence of Obesity and Severe Obesity Among Adults: United States, 2017–2018. NCHS Data Brief. 2020;(360):1–8. Epub 2020/06/04. . 32487284

[2] Must A, Spadano J, Coakley EH, Field AE, Colditz G, Dietz WH. The disease burden associated with overweight and obesity. JAMA. 1999;282(16):1523–9. Epub 1999/11/05. doi: 10.1001/jama.282.16.1523 . 10546691

[3] Hamman RF, Wing RR, Edelstein SL, Lachin JM, Bray GA, Delahanty L, et al. Effect of weight loss with lifestyle intervention on risk of diabetes. Diabetes Care. 2006;29(9):2102–7. Epub 2006/08/29. doi: 10.2337/dc06-0560 ; PubMed Central PMCID: PMC1762038. 16936160 PMC1762038

[4] Martin CB, Herrick KA, Sarafrazi N, Ogden CL. Attempts to Lose Weight Among Adults in the United States, 2013–2016. NCHS Data Brief. 2018;(313):1–8. Epub 2018/07/26. . 30044214

[5] Anderson JW, Konz EC, Frederich RC, Wood CL. Long-term weight-loss maintenance: a meta-analysis of US studies. Am J Clin Nutr. 2001;74(5):579–84. Epub 2001/10/31. doi: 10.1093/ajcn/74.5.579 . 11684524

[6] Nordmo M, Danielsen YS, Nordmo M. The challenge of keeping it off, a descriptive systematic review of high-quality, follow-up studies of obesity treatments. Obes Rev. 2020;21(1):e12949. Epub 2019/11/02. doi: 10.1111/obr.12949 . 31675146

[7] Korkeila M, Rissanen A, Kaprio J, Sorensen TI, Koskenvuo M. Weight-loss attempts and risk of major weight gain: a prospective study in Finnish adults. Am J Clin Nutr. 1999;70(6):965–75. Epub 1999/12/03. doi: 10.1093/ajcn/70.6.965 . 10584040

[8] Chae JS, Kang R, Kwak JH, Paik JK, Kim OY, Kim M, et al. Supervised exercise program, BMI, and risk of type 2 diabetes in subjects with normal or impaired fasting glucose. Diabetes Care. 2012;35(8):1680–5. Epub 2012/06/13. doi: 10.2337/dc11-2074 ; PubMed Central PMCID: PMC3402273. 22688549 PMC3402273

[9] Manson JE, Nathan DM, Krolewski AS, Stampfer MJ, Willett WC, Hennekens CH. A prospective study of exercise and incidence of diabetes among US male physicians. JAMA. 1992;268(1):63–7. Epub 1992/07/01. . 1608115

[10] Hertzmark E, Pazaris M, Spiegelman D. The SAS MEDIATE Macro 2018 [cited 2022 Jun 10]. Available from: https://cdn1.sph.harvard.edu/wp-content/uploads/sites/271/2012/08/mediate.pdf.

[11] Skender ML, Goodrick GK, Del Junco DJ, Reeves RS, Darnell L, Gotto AM, et al. Comparison of 2-year weight loss trends in behavioral treatments of obesity: diet, exercise, and combination interventions. J Am Diet Assoc. 1996;96(4):342–6. Epub 1996/04/01. doi: 10.1016/S0002-8223(96)00096-X . 8598434

[12] Foright RM, Presby DM, Sherk VD, Kahn D, Checkley LA, Giles ED, et al. Is regular exercise an effective strategy for weight loss maintenance? Physiol Behav. 2018;188:86–93. Epub 2018/02/01. doi: 10.1016/j.physbeh.2018.01.025 ; PubMed Central PMCID: PMC5929468. 29382563 PMC5929468

[13] Marinilli Pinto A, Gorin AA, Raynor HA, Tate DF, Fava JL, Wing RR. Successful weight-loss maintenance in relation to method of weight loss. Obesity (Silver Spring, Md). 2008;16(11):2456–61. Epub 2008/08/23. doi: 10.1038/oby.2008.364 ; PubMed Central PMCID: PMC2666007. 18719680 PMC2666007

[14] Catenacci VA, Pan Z, Ostendorf D, Brannon S, Gozansky WS, Mattson MP, et al. A randomized pilot study comparing zero-calorie alternate-day fasting to daily caloric restriction in adults with obesity. Obesity (Silver Spring, Md). 2016;24(9):1874–83. Epub 2016/08/30. doi: 10.1002/oby.21581 ; PubMed Central PMCID: PMC5042570. 27569118 PMC5042570

[15] Liu D, Huang Y, Huang C, Yang S, Wei X, Zhang P, et al. Calorie restriction with or without time-restricted eating in weight loss. N Engl J Med. 2022;386 (16):1495–1504. doi: 10.1056/NEJMoa2114833 . 35443107

[16] Greenway FL. Physiological adaptations to weight loss and factors favouring weight regain. Int J Obes (Lond). 2015;39(8):1188–96. Epub 2015/04/22. doi: 10.1038/ijo.2015.59 ; PubMed Central PMCID: PMC4766925. 25896063 PMC4766925

[17] Dulloo AG, Miles-Chan JL, Schutz Y. Collateral fattening in body composition autoregulation: its determinants and significance for obesity predisposition. Eur J Clin Nutr. 2018;72(5):657–64. Epub 2018/03/22. doi: 10.1038/s41430-018-0138-6 ; PubMed Central PMCID: PMC5945583. 29559726 PMC5945583

[18] Leibel RL, Rosenbaum M, Hirsch J. Changes in energy expenditure resulting from altered body weight. N Engl J Med. 1995;332(10):621–8. Epub 1995/03/09. doi: 10.1056/NEJM199503093321001 . 7632212

[19] Lean ME, Malkova D. Altered gut and adipose tissue hormones in overweight and obese individuals: cause or consequence? Int J Obes (Lond). 2016;40(4):622–32. Epub 2015/10/27. doi: 10.1038/ijo.2015.220 ; PubMed Central PMCID: PMC4827002. 26499438 PMC4827002

[20] Redman LM, Heilbronn LK, Martin CK, de Jonge L, Williamson DA, Delany JP, et al. Metabolic and behavioral compensations in response to caloric restriction: implications for the maintenance of weight loss. PLoS ONE. 2009;4(2):e4377. Epub 2009/02/10. doi: 10.1371/journal.pone.0004377 ; PubMed Central PMCID: PMC2634841. 19198647 PMC2634841

[21] Thompson D, Karpe F, Lafontan M, Frayn K. Physical activity and exercise in the regulation of human adipose tissue physiology. Physiol Rev. 2012;92(1):157–91. Epub 2012/02/03. doi: 10.1152/physrev.00012.2011 . 22298655

[22] Chaput JP, Klingenberg L, Rosenkilde M, Gilbert JA, Tremblay A, Sjödin A. Physical activity plays an important role in body weight regulation. J Obes. 2011;2011. Epub 2010/09/18. doi: 10.1155/2011/360257 ; PubMed Central PMCID: PMC2931400. 20847894 PMC2931400

[23] Son JW, Lee SS, Kim SR, Yoo SJ, Cha BY, Son HY, et al. Low muscle mass and risk of type 2 diabetes in middle-aged and older adults: findings from the KoGES. Diabetologia. 2017;60(5):865–72. Epub 2017/01/20. doi: 10.1007/s00125-016-4196-9 . 28102434

[24] Hocking S, Samocha-Bonet D, Milner KL, Greenfield JR, Chisholm DJ. Adiposity and insulin resistance in humans: the role of the different tissue and cellular lipid depots. Endocr Rev. 2013;34(4):463–500. Epub 2013/04/04. doi: 10.1210/er.2012-1041 . 23550081

[25] Chaston TB, Dixon JB, O’Brien PE. Changes in fat-free mass during significant weight loss: a systematic review. Int J Obes (Lond). 2007;31(5):743–50. Epub 2006/11/01. doi: 10.1038/sj.ijo.0803483 . 17075583

[26] Verheggen RJ, Maessen MF, Green DJ, Hermus AR, Hopman MT, Thijssen DH. A systematic review and meta-analysis on the effects of exercise training versus hypocaloric diet: distinct effects on body weight and visceral adipose tissue. Obes Rev. 2016;17(8):664–90. Epub 2016/05/24. doi: 10.1111/obr.12406 . 27213481

[27] Duncan GE, Perri MG, Theriaque DW, Hutson AD, Eckel RH, Stacpoole PW. Exercise training, without weight loss, increases insulin sensitivity and postheparin plasma lipase activity in previously sedentary adults. Diabetes Care. 2003;26(3):557–62. Epub 2003/03/01. doi: 10.2337/diacare.26.3.557 . 12610001

[28] Ahern AL, Wheeler GM, Aveyard P, Boyland EJ, Halford JCG, Mander AP, et al. Extended and standard duration weight-loss programme referrals for adults in primary care (WRAP): a randomised controlled trial. Lancet (London, England). 2017;389(10085):2214–25. Epub 2017/05/10. doi: 10.1016/S0140-6736(17)30647-5 ; PubMed Central PMCID: PMC5459752. 28478041 PMC5459752

[29] Gosselin C, Cote G. Weight loss maintenance in women two to eleven years after participating in a commercial program: a survey. BMC Womens Health. 2001;1:2. Epub 2001/09/05. doi: 10.1186/1472-6874-1-2 ; PubMed Central PMCID: PMC48152. 11532203 PMC48152

[30] Finley CE, Barlow CE, Greenway FL, Rock CL, Rolls BJ, Blair SN. Retention rates and weight loss in a commercial weight loss program. Int J Obes (Lond). 2007;31(2):292–8. Epub 2006/06/07. doi: 10.1038/sj.ijo.0803395 . 16755283

[31] Bray GA. Use and abuse of appetite-suppressant drugs in the treatment of obesity. Ann Intern Med. 1993;119(7 Pt 2):707–13. Epub 1993/10/01. doi: 10.7326/0003-4819-119-7_part_2-199310011-00016 . 8363202

[32] Hendricks EJ. Off-label drugs for weight management. Diabetes Metab Syndr Obes. 2017;10:223–34. Epub 2017/06/28. doi: 10.2147/DMSO.S95299 ; PubMed Central PMCID: PMC5473499. 28652791 PMC5473499

[33] Yanovski SZ, Yanovski JA. Long-term drug treatment for obesity: a systematic and clinical review. JAMA. 2014;311(1):74–86. Epub 2013/11/16. doi: 10.1001/jama.2013.281361 ; PubMed Central PMCID: PMC3928674. 24231879 PMC3928674

[34] Prescription Medications to Treat Overweight & Obesity website: National Institute of Diabetes and Digestive and Kidney Diseases; 2021 [updated 2021 Jun; cited 2022 Jun 10]. Available from: https://www.niddk.nih.gov/health-information/weight-management/prescription-medications-treat-overweight-obesity#replace.

[35] Enríquez Guerrero A, San Mauro Martín I, Garicano Vilar E, Camina Martín MA. Effectiveness of an intermittent fasting diet versus continuous energy restriction on anthropometric measurements, body composition and lipid profile in overweight and obese adults: a meta-analysis. Eur J Clin Nutr. 2021;75(7):1024–39. Epub 2020/12/10. doi: 10.1038/s41430-020-00821-1 . 33293678

[36] Seimon RV, Roekenes JA, Zibellini J, Zhu B, Gibson AA, Hills AP, et al. Do intermittent diets provide physiological benefits over continuous diets for weight loss? A systematic review of clinical trials. Mol Cell Endocrinol. 2015;418 Pt 2:153–72. Epub 2015/09/20. doi: 10.1016/j.mce.2015.09.014 . 26384657

[37] Knowler WC, Fowler SE, Hamman RF, Christophi CA, Hoffman HJ, Brenneman AT, et al. 10-year follow-up of diabetes incidence and weight loss in the Diabetes Prevention Program Outcomes Study. Lancet (London, England). 2009;374(9702):1677–86. Epub 2009/11/03. doi: 10.1016/S0140-6736(09)61457-4 ; PubMed Central PMCID: PMC3135022. 19878986 PMC3135022

[38] Tuomilehto J, Schwarz P, Lindström J. Long-term benefits from lifestyle interventions for type 2 diabetes prevention: time to expand the efforts. Diabetes Care. 2011;34 Suppl 2(Suppl 2):S210–4. Epub 2011/05/06. doi: 10.2337/dc11-s222 ; PubMed Central PMCID: PMC3632163. 21525457 PMC3632163

[39] Santos I, Sniehotta FF, Marques MM, Carraça EV, Teixeira PJ. Prevalence of personal weight control attempts in adults: a systematic review and meta-analysis. Obes Rev. 2017;18(1):32–50. Epub 2016/09/23. doi: 10.1111/obr.12466 ; PubMed Central PMCID: PMC5215364. 27653242 PMC5215364

[40] Lemon SC, Rosal MC, Zapka J, Borg A, Andersen V. Contributions of weight perceptions to weight loss attempts: differences by body mass index and gender. Body Image 2009;6(2):90–6. Epub 2009/02/04. doi: 10.1016/j.bodyim.2008.11.004 ; PubMed Central PMCID: PMC2692706. 19188102 PMC2692706

[41] Sares-Jäske L, Knekt P, Männistö S, Lindfors O, Heliövaara M. Self-report dieting and long-term changes in body mass index and waist circumference. Obes Sci Pract. 2019;5(4):291–303. Epub 2019/08/28. doi: 10.1002/osp4.336 ; PubMed Central PMCID: PMC6700513. 31452914 PMC6700513

[42] Dulloo AG, Jacquet J, Montani JP, Schutz Y. How dieting makes the lean fatter: from a perspective of body composition autoregulation through adipostats and proteinstats awaiting discovery. Obes Rev. 2015;16 Suppl 1:25–35. Epub 2015/01/24. doi: 10.1111/obr.12253 . 25614201

[43] Woo R, Pi-Sunyer FX. Effect of increased physical activity on voluntary intake in lean women. Metab Clin Exp. 1985;34(9):836–41. Epub 1985/09/01. doi: 10.1016/0026-0495(85)90108-8 . 4033425

[44] Donnelly JE, Hill JO, Jacobsen DJ, Potteiger J, Sullivan DK, Johnson SL, et al. Effects of a 16-month randomized controlled exercise trial on body weight and composition in young, overweight men and women: the Midwest Exercise Trial. Arch Intern Med. 2003;163(11):1343–50. Epub 2003/06/11. doi: 10.1001/archinte.163.11.1343 . 12796071

[45] Cava E, Yeat NC, Mittendorfer B. Preserving Healthy Muscle during Weight Loss. Adv Nutr. 2017;8(3):511–9. Epub 2017/05/17. doi: 10.3945/an.116.014506 ; PubMed Central PMCID: PMC5421125. 28507015 PMC5421125

[46] Jensen MD, Ryan DH, Apovian CM, Ard JD, Comuzzie AG, Donato KA, et al. 2013 AHA/ACC/TOS guideline for the management of overweight and obesity in adults: a report of the American College of Cardiology/American Heart Association Task Force on Practice Guidelines and The Obesity Society. Circulation. 2014;129(25 Suppl 2):S102–38. Epub 2013/11/14. doi: 10.1161/01.cir.0000437739.71477.ee ; PubMed Central PMCID: PMC5819889. 24222017 PMC5819889

