# Supplementary material for: Weight loss strategies, weight change, and type 2 diabetes in US health professionals: A cohort study
Source: PLoS Med. 2022 Sep 27;19(9):e1004094. doi: 10.1371/journal.pmed.1004094 (PMC9514663; doi:10.1371/journal.pmed.1004094)
Supplement: S1 Text — (DOCX) [file pmed.1004094.s002.docx]

**S1 Text. Study protocol.**

The following protocol was originally submitted to the review board of the Brigham and Women’s Hospital and the Harvard T.H. Chan School of Public Health before analyzing the data.

**Title**

Weight Loss Strategies, Weight Change, and Type 2 Diabetes

**Study Population(s)**

NHS1, NHS2, HPFS

**Background**

Obesity is one of the most common and serious public health problems in the U.S. In 2015-2016, 39.8% of U.S. adults had obesity [1], which is a predominant risk factor for multiple chronic diseases, especially type 2 diabetes (T2D) [2]. Weight control or management is one of the primary strategies for diabetes prevention and management. In 2013-2016, it was estimated that nearly half of U.S. adults tried to lose weight [3], mainly through lifestyle modification (diet, exercise), pharmacotherapy, or surgery. On average, the risk of diabetes is reduced by 16% per kilogram weight loss [4]. However, it is notoriously challenging to maintain weight loss in a long term [5]. Some studies found that weight-loss methods may have differential trajectory of weight regain [6,7], which might thereby exert different impacts on the risk of T2D. However, to date, no study has comprehensively examined multiple methods of weight control in relation to T2D risk. In addition, the effects may vary by baseline weight and abdominal fat mass. It is known that fat mass is detrimental but fat-free mass is protective for T2D [8,9]. Studies have indicated that people with normal weight were more likely to lose fat-free mass and regain fat than those who were overweight or obese [10], although whether the effects of different weight-loss methods differ by baseline weight has not been examined.

To fill these knowledge gaps, the current study aimed to investigate the association of weight control methods (diet, exercise, fasting, pills, and commercial weight loss programs) with T2D risk and weight change. We also evaluated these associations according to baseline body mass index (BMI) and waist-hip ratio (WHR), in three large prospective cohort studies of U.S. men and women.

**Statement of Hypothesis**

Various methods of weight control may be differentially associated with long-term weight change and risk of T2D. The association may be modified by baseline BMI and abdominal obesity.

**Study Design**

Prospective cohort study.
The baseline was 1988 for HPFS and NHS, and 1989 for NHS II; the follow-up period for T2D and weight change was 20 years and 6 years from 1992 for HPFS and NHS, and 1993 for NHS II.

T2D
Baseline Exclusions
1. Only baseline questionnaire available
2. Previous diagnosis of cancer, cardiovascular disease (angina pectoris, coronary artery bypass grafting, myocardial infarction, or stroke), or diabetes
3. Unknown methods of weight control
4. Unintentional weight loss
5. Weight control via gastric surgery/intestinal bypass or other unknown methods
6. Unknown baseline BMI
7. Unknown diagnosis date of T2D/death

Censor During Follow-up
1. Incident T2D, death, cancer, or cardiovascular disease
2. Lost to follow-up

Weight change
Baseline Exclusions
1. Items 2-6 mentioned above
2. Aged 65+ years at baseline
3. Weights in all follow-up cycles were not available

Censor During Follow-up
1. Aged 65+ years at follow-up
2. Pregnant at follow-up
3. Incident T2D, death, cancer, or cardiovascular disease

**Exposure(s)**

Methods of weight control used most recently to lose 10 or more pounds intentionally within last 4 years in 1992 (HPFS and NHS) or 1993 (NHS II)

1. Did not lose 10 or more pounds (reference)
2. Low-calorie diet only
3. Increased exercise only
4. Low-calorie diet and increased exercise
5. Skipped meals/fasted with or without low-calorie diet/increased exercise
6. Commercial weight loss program with or without low-calorie diet/increased exercise
7. Diet pills with or without low-calorie diet/increased exercise
8. Other (choose two or three methods among skipped meals/fasted, commercial weight loss program, and diet pills, with or without low-calorie diet/increased exercise)

The options of weight control methods in the 1992 questionnaire included ‘did not lose 10 or more pounds’, ‘weight loss was unintentional (e.g., illness, unusual stress, depression)’, ‘low-calorie diet’, ‘skipped meals/fasted’, ‘increased exercise’, ‘diet pills’, ‘commercial weight loss program’, ‘gastric surgery/intestinal bypass’, and ‘other’. In the 1993 questionnaire, they were extended with ‘low fat diet’, ‘decreased alcohol intake’, and ‘resumed/increased smoking’. To facilitate analyses and interpretation, these multiple-choice items were reclassified and unified into mutually exclusive ones as mentioned above according to their frequency.

**Outcome(s)**

1. Incident T2D diagnosed in 1992-2012 (HPFS and NHS), and 1993-2013 (NHS II)
2. Weight in 1992-1998 (HPFS and NHS), and 1993-1999 (NHS II)

Information on newly diagnosed T2D and weight were updated biennially through self-administered questionnaires. Once participants reported a physician diagnosis of diabetes, they were mailed a validated supplementary questionnaire to confirm the diagnosis.

**Covariates**

1. Age (in months, continuous)
2. Gender (male or female)
3. Cohort (HPFS, NHS or NHS II)
4. Baseline BMI (continuous; weight in kilograms divided by height in meters squared)
5. Abdominal obesity (yes or no; WHR>0.90 for men and >0.85 for women)
6. Ethnicity (white or not)
7. Alcohol intake (0, <5.0, 5.0 to 9.9, 10.0 to 14.9, 15.0 to 29.9, or >30.0 g per day)
8. Smoking status (never smoker, past smoker, or current smoker in HPFS, never smoker, former smoker, current smoker: 1-14, 15-24, or ≥25 cigarettes/day in NHS and NHS II)
9. Multivitamin use (yes or no)
10. Television watching duration (0-1h, 2-5h, 6-10h, 11-20h, or 21+h)
11. Menopausal status (premenopausal or postmenopausal, never, past, or current menopausal hormone use in NHS and NHS II)
12. Total energy intake (in quintiles)
13. Alternative Healthy Eating Index score (AHEI, in quintiles)
14. Physical activity quantified as metabolic equivalent tasks (METs, in quintiles)
15. History of hypertension (yes or no)
16. History of hypercholesterolemia (yes or no)
17. Family history of diabetes (yes or no)

Lifestyle and medical information were updated biennially from the very beginning of the studies (1986 for HPFS, 1976 for NHS, and 1989 for NHS II). A tailored semiquantitative food frequency questionnaire and a validated questionnaire regarding time spent on up to 10 recreational activities were administered every 2-4 years since 1986 (HPFS and NHS) and 1991 (NHS II), in which AHEI score, total energy intake, and METS were derived.

In this study, information on some of the covariates, including age, baseline BMI, alcohol intake, smoking status, multivitamin use, menopausal status, and METs were collected from the baseline questionnaires. Others were substituted with values from the nearest-neighbor questionnaires.

**Statistical Analysis**

Data from the three cohorts were pooled to maximize statistical power. Person-time for each participant was counted from the return of the baseline questionnaire to the date of T2D diagnosis, death, last return of a valid follow-up questionnaire, or the end of follow-up (June 2012 for the HPFS and the NHS; June 2013 for the NHS II), whichever came first.

Cox proportional hazards model conditioned on age and follow-up cycle was applied to examine the association of methods of weight control with the incidence of T2D. Proportionality assumptions were verified by including the interaction terms between each exposure indicator and log-transformed follow-up time in the model. Generalized estimating equation model was used to assess the association between methods of weight control and subsequent weight change within 6 years. Least-squares mean of weight in each 2-year survey cycle was calculated to explore the trend of weight change.

Multiple Imputation
Multiple imputation was implemented for 5 times for covariates with missing values. Since these covariates were a mixture of continuous and categorical variables with an arbitrary missing pattern, the fully conditional specification method was used. First, time-independent ordinal (television watching duration) and nominal variables (smoking and menopausal status) were imputed through logistic regression and discriminant techniques, respectively. Variables, including methods of weight control, age, ethnicity, baseline BMI, weight cycling, multivitamin use, hypertension, hypercholesterolemia, family history of diabetes, were included as covariates in the imputation model. Then, time-dependent continuous variables, including total energy, alcohol intake, AHEI score, METs, BMI, and WHR, were imputed with predictive mean matching, before the first 4 variables were made into categories. In addition to the covariates in the time-independent imputation model, three newly filled-in variables were served as covariates in this imputation model. The beta coefficients and standard errors estimated from imputed datasets were pooled using PROC MIANALYZE in SAS.

Mediation Analyses
Given that BMI might be a mediator between methods of weight control and risk of T2D, the extent to which the association could be explained by this factor (time-varying) was evaluated using a SAS macro %MEDIATE.

Stratified Analyses
Stratified analyses were conducted by age (<65 years, ≥65 years), and baseline BMI (<25.0 kg/m^2^, 25.0-29.9 kg/m^2^, ≥30 kg/m^2^), among which participants whose BMI was below 25.0 kg/m^2^ were further stratified by baseline abdominal obesity (yes, no). Interactions were tested by a likelihood-ratio test comparing models with and without product terms between methods of weight control and stratifying variables.

Sensitivity Analyses
To test whether the association between methods of weight control and risk of T2D was consistent across time, estimates were recalculated in the first and last 10 years’ follow-up, respectively.

To reduce the possibility of reverse-causation, we excluded participants who were diagnosed T2D in the first 4-year follow-up.

All P values were two-sided. Data were analyzed using SAS software, version 9.4 (SAS Institute).

References
1. Hales CM, Carroll MD, Fryar CD, Ogden CL. Prevalence of obesity among adults and youth: United States, 2015–2016. NCHS data brief, no 288. Hyattsville, MD: National Center for Health Statistics. 2017.
2. Must A, Spadano J, Coakley EH, Field AE, Colditz G, Dietz WH. The disease burden associated with overweight and obesity. JAMA. 1999;282(16):1523-1529.
3. Martin CB, Herrick KA, Sarafrazi N, Ogden CL. Attempts to lose weight among adults in the United States, 2013–2016. NCHS Data Brief, no 313. Hyattsville, MD: National Center for Health Statistics. 2018.
4. Hamman RF, Wing RR, Edelstein SL, Lachin JM, et al. Effect of weight loss with lifestyle intervention on risk of diabetes. Diabetes Care. 2006;29(9):2102-7.
5. Anderson JW, Konz EC, Frederich RC, Wood CL. Long-term weight-loss maintenance: a meta-analysis of US studies. Am J Clin Nutr. 2001;74(5):579–584.
6. Marinilli Pinto A, Gorin AA, Raynor HA, Tate DF, Fava JL, Wing RR. Successful weight-loss maintenance in relation to method of weight loss. Obesity (Silver Spring). 2008;16(11):2456-61.
7. Dombrowski SU, Knittle K, Avenell A, Araújo-Soares V, Sniehotta FF. Long term maintenance of weight loss with non-surgical interventions in obese adults: systematic review and meta-analyses of randomised controlled trials. BMJ. 2014;348:g2646.
8. Lee DH, Keum N, Hu FB, et al. Comparison of the association of predicted fat mass, body mass index, and other obesity indicators with type 2 diabetes risk: two large prospective studies in US men and women. Eur J Epidemiol. 2018;33(11):1113-1123.
9. Son JW, Lee SS, Kim SR, et al. Low muscle mass and risk of type 2 diabetes in middle-aged and older adults: findings from the KoGES. Diabetologia. 2017;60(5):865-872.
10. Cava E, Yeat NC, Mittendorfer B. Preserving Healthy Muscle during Weight Loss. Adv Nutr. 2017;8(3):511-519.

**Modifications to the original analysis plan for the current analyses**

The current analyses are consistent with those that were planned in the original analysis plan described above except for the modifications presented below.

1. **Length of follow-up**: After we submitted the study protocol, data of two more circles of follow-up (2014 and 2016 for NHS/HPFS, 2015 and 2017 for NHSII) were updated, we therefore set June 2016 and June 2017 as the new end of follow-up for NHS/HPFS and NHSII, respectively.
2. **Inclusion and exclusion criteria**: Three more detailed exclusion criteria were added: (1) participants who lost less than 10 lbs (N=37,207) were separated from the reference group (participants who did not lose 10+ lbs) and excluded so that the new reference group (participants who did not attempt to lose weight) can be more homogeneous; (2) participants with missing age (N=39) were excluded; (3) participants who were pregnant in 1989 (N=2600) were excluded because their baseline body weight assessed in the 1989 questionnaire were not typical. In addition, to avoid wasting data, participants who were excluded from the T2D analysis because of only answering the 1992/1993 questionnaire were included in the weight change analysis if they contributed valid body weight assessments in that year (N=573).
3. **Multivariate adjustment**: As the editors and reviewers suggested, the dichotomous ethnicity (white or not) has been reclassified into four categories: white, African American, Asian, and other; baseline body weight rather than baseline body mass index was adjusted for in the weight change analyses. To reduce the potential residual confounding effect, we adjusted for continuous waist circumference rather than dichotomous abdominal obesity. Gender and menopausal status were not adjusted for as planned because they have collinearity with the variable “cohort” (HPFS-male, NHS-female, NHSII-female).
4. **Stratified and sensitivity analyses**: Firstly, we planned to conduct a stratified analysis by age (<65 years, ≥65 years), but the number of old participants in some weight loss strategy groups were too small to get a robust estimate, therefore we repeated the main analysis restricted to participants who were less than 65 years old instead. Secondly, to test whether the association between methods of weight control and risk of T2D was consistent across time, estimates were planned to be recalculated in the first and last 10 years’ follow-up, respectively. We later found a better method to deal with this problem--a cubic spline regression model was fitted to delineate the trajectory of hazard ratios over the follow-up duration. Thirdly, three more sensitivity analyses were added as suggested by the co-authors: (1) to alleviate the concern that the body weight assessments in 1988 (NHS/HPFS) or 1989 (NHSII) may misclassify the long-term weight status before 1988/1989, we redefined individuals who were consistently lean (BMI was less than 25 kg/m^2^ at each biennial follow-up from the initiation of the cohorts to 1988/1989) as the baseline lean group, and the same algorithm was used to define the overweight and obese groups; (2) we used maximum BMI enquired before 1992/1993 (1972-1992 for HPFS, 1976-1992 for NHS, 1989-1993 for NHSII) to define the obesity status; (3) we included participants who skipped the weight loss strategy question into the reference group.
