# Supplementary material for: Weight loss strategies, weight change, and type 2 diabetes in US health professionals: A cohort study
Source: PLoS Med. 2022 Sep 27;19(9):e1004094. doi: 10.1371/journal.pmed.1004094 (PMC9514663; doi:10.1371/journal.pmed.1004094)
Supplement: S2 Text — (DOCX) [file pmed.1004094.s003.docx]

**S2 Text. The confirmation of self-reported type 2 diabetes.**

For cases diagnosed before 1998, type 2 diabetes (T2D) was confirmed if participants met at least one of the following National Diabetes Data Group criteria [1]: (1) an elevated glucose concentration (fasting plasma glucose of 7.8 mmol/l, random plasma glucose of 11.1 mmol/l, or plasma glucose 11.1 mmol/l after an oral glucose load), and at least one symptom related to diabetes (excessive thirst, polyuria, weight loss, or hunger); (2) no symptoms, but elevated glucose concentrations on two occasions; and (3) treatment with insulin or other hypoglycemic medication. For cases of T2D identified after 1998, the cut off point for elevated fasting plasma glucose concentrations was lowered to 7.0 mmol/l according to the American Diabetes Association criteria. We further considered HbA1c ≥6.5% in the diagnosis criteria for confirming T2D cases identified after January, 2010 [2].

In previous validation studies, there was a strong correlation (r=0.97) between self-reported and measured body weight [3], and 97% and 98% of questionnaire-confirmed T2D in the HPFS and NHS were re-confirmed by blinded medical record review, respectively [4,5].

**References**

[1] National Diabetes Data Group (1979) Classification and diagnosis of diabetes mellitus and other categories of glucose intolerance. Diabetes 28(12):1039-1057. https://doi/org/[10.2337/diab.28.12.1039](https://doi.org/10.2337/diab.28.12.1039)

[2] American Diabetes Association (2010) Standards of medical care in diabetes--2010. Diabetes Care 33 Suppl 1(Suppl 1):S11-S61. https://doi/org/[10.2337/dc10-S011](https://doi.org/10.2337/dc10-s011)

[3] Troy LM, Hunter DJ, Manson JE, Colditz GA, Stampfer MJ, Willett WC (1995) The validity of recalled weight among younger women. Int J Obes Relat Metab Disord 19(8):570-572.

[4] Manson JE, Colditz GA, Stampfer MJ, et al (1991) A prospective study of maturity-onset diabetes mellitus and risk of coronary heart disease and stroke in women. Arch Intern Med 151(6):1141-1147.

[5] Hu FB, Leitzmann MF, Stampfer MJ, Colditz GA, Willett WC, Rimm EB (2001) Physical activity and television watching in relation to risk for type 2 diabetes mellitus in men. Arch Intern Med 161(12):1542-1548. https://doi/org/[10.1001/archinte.161.12.1542](https://doi.org/10.1001/archinte.161.12.1542)
