# Supplementary material for: Weight loss strategies, weight change, and type 2 diabetes in US health professionals: A cohort study
Source: PLoS Med. 2022 Sep 27;19(9):e1004094. doi: 10.1371/journal.pmed.1004094 (PMC9514663; doi:10.1371/journal.pmed.1004094)
Supplement: S3 Text — (DOCX) [file pmed.1004094.s004.docx]

**S3 Text. Multiple imputation of the covariates.**

Multiple imputation was implemented for 5 times for variables with missing values, including television watching hours, smoking and menstrual status and hormone use, waist circumference, alcohol intake, total energy intake, Alternative Healthy Eating Index (AHEI) score, metabolic equivalent tasks (METs), and time-dependent body mass index (BMI) from 1992/1993 to 2014/2015. Details are presented in the table below. Since they were a mixture of continuous and categorical variables with an arbitrary missing pattern, the fully conditional specification method was used for imputation. First, time-independent ordinal (television watching duration), nominal (smoking and menstrual status and hormone use), and continuous variables (waist circumference, alcohol intake, total energy intake, AHEI score, METs) were imputed through logistic regression, discriminant techniques, and regression, respectively, before the continuous variables were made into categories. Variables, including methods of weight control, age, ethnicity, baseline BMI, multivitamin use, hypertension, hypercholesterolemia, family history of diabetes, were included as covariates in the imputation model. Then, time-dependent BMI was imputed using predictive mean matching, with all above-mentioned variables, including the imputed ones, as covariates. The beta coefficients and standard errors estimated from imputed datasets were pooled using PROC MIANALYZE in SAS.

| **Variables** | **Percentages of Missing Data (%)** | |
| --- | --- | --- |
|  | **Weight Change Analyses** | **T2D Analyses** |
| METs | 0.3 | 0.4 |
| Smoking status | 0.5 | 0.6 |
| Menstrual status and hormone use | 0.5 | 1.1 |
| Television watching duration | 4.0 | 3.7 |
| AHEI score | 11.3 | 11.2 |
| Total calorie intake | 11.3 | 11.2 |
| Alcohol intake | 11.3 | 11.2 |
| BMI in 1992/1993 | - | 7.2 |
| BMI in 1994/1995 | - | 7.3 |
| BMI in 1996/1997 | - | 7.4 |
| BMI in 1998/1999 | - | 9.8 |
| BMI in 2000/2001 | - | 10.8 |
| BMI in 2002/2003 | - | 12.1 |
| BMI in 2004/2005 | - | 14.1 |
| BMI in 2006/2007 | - | 18.6 |
| BMI in 2008/2009 | - | 20.4 |
| BMI in 2010/2011 | - | 21.8 |
| BMI in 2012/2013 | - | 26.9 |
| BMI in 2014/2015 | - | 29.4 |
| Baseline waist circumference | 43.3 | 41.7 |
