# Supplementary material for: Weight loss strategies, weight change, and type 2 diabetes in US health professionals: A cohort study
Source: PLoS Med. 2022 Sep 27;19(9):e1004094. doi: 10.1371/journal.pmed.1004094 (PMC9514663; doi:10.1371/journal.pmed.1004094)
Supplement: S1 Table — (DOCX) [file pmed.1004094.s005.docx]

**S1 Table. Components of the weight loss strategies in the type 2 diabetes analyses.**

| **No.** | **Weight Loss Strategies** | **Components** | **HPFS** | **NHS** | **NHS II** |
| --- | --- | --- | --- | --- | --- |
|  |  |  | **n (%)** | **n (%)** | **n (%)** |
| 1 | Reference | Did not attempt to lose weight | 6,468 (100) | 10,318 (100) | 12,193 (100) |
| 2 | LCD | LCD | 1,631 (100) | 4,647 (100) | 3,694 (100) |
| 3 | Exercise | Increased exercise | 806 (100) | 819 (100) | 6,383 (100) |
| 4 | LCD & Exercise | LCD & Exercise | 2,914 (100) | 8,401 (100) | 10,656 (100) |
| 5 | Fasting | Skipped meals/fasted (fasting for short) | 456 (22.1) | 385 (20.0) | 869 (16.0) |
|  |  | Fasting & LCD | 317 (15.3) | 423 (22.0) | 1,014 (18.6) |
|  |  | Fasting & Exercise | 563 (27.3) | 308 (16.0) | 1,164 (21.4) |
|  |  | Fasting & LCD & Exercise | 730 (35.3) | 807 (42.0) | 2,398 (44.0) |
| 6 | CWLP | CWLP | 338 (46.2) | 3,720 (47.6) | 3,636 (30.2) |
|  |  | CWLP & LCD | 98 (13.4) | 949 (12.2) | 1,419 (11.8) |
|  |  | CWLP & Exercise | 118 (16.1) | 1,302 (16.7) | 2,685 (22.3) |
|  |  | CWLP & LCD & Exercise | 177 (24.2) | 1,836 (23.5) | 4,303 (35.7) |
| 7 | Pill | Diet pills | 6 (18.8) | 41 (12.0) | 169 (16.0) |
|  |  | Pill & LCD | 7 (21.9) | 101 (29.5) | 180 (17.0) |
|  |  | Pill & Exercise | 5 (15.6) | 25 (7.3) | 239 (22.6) |
|  |  | Pill & LCD & Exercise | 14 (43.8) | 175 (51.2) | 471 (44.5) |
| 8 | FCP | Fasting & CWLP | 12 (7.0) | 66 (8.7) | 156 (5.4) |
|  |  | Fasting & CWLP & LCD | 30 (17.5) | 89 (11.7) | 272 (9.5) |
|  |  | Fasting & CWLP & Exercise | 13 (7.6) | 53 (6.9) | 191 (6.7) |
|  |  | Fasting & CWLP & LCD & Exercise | 50 (29.2) | 187 (24.5) | 638 (22.2) |
|  |  | Fasting & Pill | 14 (8.2) | 33 (4.3) | 130 (4.5) |
|  |  | Fasting & Pill & LCD | 10 (5.8) | 43 (5.6) | 150 (5.2) |
|  |  | Fasting & Pill & Exercise | 12 (7.0) | 30 (3.9) | 156 (5.4) |
|  |  | Fasting & Pill & LCD & Exercise | 16 (9.4) | 75 (9.8) | 406 (14.2) |
|  |  | CWLP & Pill | 1 (0.6) | 16 (2.1) | 70 (2.4) |
|  |  | CWLP & Pill & LCD | 3 (1.8) | 27 (3.5) | 62 (2.2) |
|  |  | CWLP & Pill & Exercise | 1 (0.6) | 17 (2.2) | 84 (2.9) |
|  |  | CWLP & Pill & LCD & Exercise | 2 (1.2) | 61 (8.0) | 207 (7.2) |
|  |  | Fasting & CWLP & Pill | 1 (0.6) | 5 (0.7) | 31 (1.1) |
|  |  | Fasting & CWLP & Pill & LCD | 0 (0) | 14 (1.8) | 58 (2.0) |
|  |  | Fasting & CWLP & Pill & Exercise | 2 (1.2) | 3 (0.4) | 45 (1.6) |
|  |  | Fasting & CWLP & Pill & LCD & Exercise | 4 (2.3) | 44 (5.8) | 212 (7.4) |
| 9 | Total |  | 14,819 (100) | 35,020 (100) | 54,341 (100) |

**Abbreviations**: CWLP, commercial weight loss program; FCP, select at least two strategies among fasting, CWLP, and pill ; HPFS, Health Professionals Follow-up Study; LCD, low-calorie diet; n, number; NHS, Nurses’ Health Study.
