# Supplementary material for: Weight loss strategies, weight change, and type 2 diabetes in US health professionals: A cohort study
Source: PLoS Med. 2022 Sep 27;19(9):e1004094. doi: 10.1371/journal.pmed.1004094 (PMC9514663; doi:10.1371/journal.pmed.1004094)
Supplement: S2 Table — (DOCX) [file pmed.1004094.s006.docx]

**S2 Table. Data source of the covariates.**

| **Covariates** | **Questionnaires** | | |
| --- | --- | --- | --- |
|  | **HPFS** | **NHS** | **NHSII** |
| Age | 1988 | | 1989 |
| Body weight |  |  |  |
| Body mass index |  |  |  |
| Smoking status |  |  |  |
| Multivitamin use |  |  |  |
| Physical activity |  |  |  |
| History of hypertension | 1992 | | 1993 |
| History of hypercholesterolemia |  |  |  |
| Alcohol intake | 1986 | | 1991 |
| Total energy intake |  |  |  |
| Diet quality |  |  |  |
| Ethnicity | 1986 | 1992 | 1989 |
| Family history of diabetes* | 1990, 1992 | 1982, 1988, 1992 | 1989 |
| Waist circumference | 1987 | 1986 | 1993 |
| Height | 1986 | 1976 | 1989 |
| Television watching duration | 1988 | 1992 | 1991 |

*Family history of diabetes had been investigated for multiple times. Once the answer was ‘yes’ in any questionnaire, the participant would be considered having a family history of diabetes. **Abbreviations**: HPFS, Health Professionals Follow-up Study; NHS, Nurses’ Health Study
