# Supplementary material for: Weight loss strategies, weight change, and type 2 diabetes in US health professionals: A cohort study
Source: PLoS Med. 2022 Sep 27;19(9):e1004094. doi: 10.1371/journal.pmed.1004094 (PMC9514663; doi:10.1371/journal.pmed.1004094)
Supplement: S3 Table — (DOCX) [file pmed.1004094.s007.docx]

**S3 Table. Age-standardized proportions of weight loss strategies by baseline body mass index in the type 2 diabetes analyses.**

| **Weight Loss Strategies (%)** | **Overall**  **(n = 75,201)** | **Baseline Body Mass Index** | | |
| --- | --- | --- | --- | --- |
|  |  | **< 25 kg/m^2^**  **(n = 37,833)** | **25 to < 30 kg/m^2^**  **(n = 24,805)** | **≥ 30 kg/m^2^**  **(n = 12,563)** |
| LCD | 13.3 | 13.3 | 12.8 | 14.5 |
| Exercise | 10.7 | 12.8 | 8.7 | 6.3 |
| LCD & Exercise | 29.2 | 32.2 | 28.1 | 22.6 |
| Fasting | 12.6 | 13.6 | 11.2 | 10.1 |
| CWLP | 27.4 | 22.3 | 31.4 | 37.1 |
| Pill | 1.9 | 1.9 | 2.0 | 1.9 |
| FCP | 5.1 | 3.9 | 5.8 | 7.5 |

Participants were limited to those who lost 4.5+ kg (10+ lbs). Values may not sum to 100% due to rounding. **Abbreviations**: CWLP, commercial weight loss program; FCP, select at least two strategies among fasting, CWLP, and pill; kg/m^2^, kilogram per square meter; LCD, low-calorie diet; n, number.
