# Supplementary material for: Weight loss strategies, weight change, and type 2 diabetes in US health professionals: A cohort study
Source: PLoS Med. 2022 Sep 27;19(9):e1004094. doi: 10.1371/journal.pmed.1004094 (PMC9514663; doi:10.1371/journal.pmed.1004094)
Supplement: S4 Table — (DOCX) [file pmed.1004094.s008.docx]

**S4 Table. Age-standardized characteristics of participants before weight loss in the type 2 diabetes analyses.**

| **Characteristic** | **Reference** | **LCD** | **Exercise** | **LCD & Exercise** | **Fasting** | **CWLP** | **Pill** | **FCP** |
| --- | --- | --- | --- | --- | --- | --- | --- | --- |
| **HPFS** | | | | | | | | |
| Participants, number | 6,468 | 1,631 | 806 | 2,914 | 2,066 | 731 | 32 | 171 |
| Age in 1992 (year) | 59.1 (9.5) | 59.2 (9.0) | 56.1 (8.6) | 56.7 (8.5) | 56.0 (8.4) | 56.1 (8.2) | 57.3 (8.3) | 53.8 (7.6) |
| Ethnicity |  |  |  |  |  |  |  |  |
| White, % | 95.8 | 96.5 | 94.6 | 97.1 | 95.6 | 96.9 | 97.8 | 95.8 |
| African American, % | 1.7 | 2.4 | 2.3 | 1.8 | 2.4 | 2.2 | 2.2 | 2.3 |
| Asian, % | 1.8 | 0.6 | 2.1 | 0.5 | 1.1 | 0.2 | 0 | 0.4 |
| Other, % | 0.6 | 0.4 | 1.0 | 0.6 | 0.9 | 0.6 | 0 | 1.5 |
| Body mass index (kg/m²) | 24.0 (2.5) | 26.8 (3.2) | 25.5 (2.7) | 26.4 (2.8) | 26.6 (3.0) | 28.1 (3.4) | 27.9 (4.3) | 28.5 (3.3) |
| Waist circumference (centimeter) | 90.9 (7.5) | 98.5 (8.7) | 94.0 (8.2) | 96.6 (8.3) | 97.6 (8.7) | 102.3 (9.7) | 100.7 (11.7) | 103.3 (10.0) |
| Smoking status |  |  |  |  |  |  |  |  |
| Never smoker, % | 52.6 | 44.1 | 47.0 | 43.4 | 45.2 | 41.0 | 41.1 | 44.4 |
| Past smoker, % | 35.2 | 42.7 | 41.6 | 47.0 | 42.6 | 49.3 | 45.6 | 41.5 |
| Current smoker, % | 9.1 | 8.5 | 8.2 | 5.2 | 8.6 | 6.6 | 11.1 | 10.1 |
| Missing, % | 3.1 | 4.7 | 3.2 | 4.4 | 3.7 | 3.0 | 2.2 | 3.9 |
| Multivitamin use, % | 38.1 | 37.4 | 42.0 | 42.3 | 41.0 | 45.7 | 36.5 | 46.9 |
| Television watching (hour) |  |  |  |  |  |  |  |  |
| 0-1, % | 5.2 | 3.2 | 4.8 | 3.6 | 4.2 | 3.4 | 2.2 | 3.8 |
| 2-5, % | 27.6 | 24.2 | 29.1 | 26.2 | 26.0 | 21.9 | 31.8 | 23.8 |
| 6-10, % | 29.2 | 29.4 | 28.6 | 29.9 | 27.7 | 29.9 | 25.0 | 29.3 |
| 11-20, % | 28.5 | 30.7 | 29.6 | 29.9 | 30.3 | 31.1 | 34.4 | 27.8 |
| 21+, % | 9.0 | 12.1 | 7.7 | 10.0 | 11.4 | 12.9 | 6.5 | 15.4 |
| Missing, % | 0.4 | 0.3 | 0.2 | 0.3 | 0.4 | 0.7 | 0 | 0 |
| Physical activity (METs-hour/week) | 19.6 (7.7, 39.8) | 14.3 (5.4, 31.7) | 28.3 (12.9, 56.0) | 24.0 (11.3, 46.2) | 17.7 (6.7, 37.3) | 15.9 (5.6, 32.2) | 17.8 (4.7, 53.6) | 14.3 (4.7, 36.6) |
| Alternative healthy eating index | 45.5 (10.9) | 46.9 (10.8) | 47.1 (10.6) | 48.9 (10.7) | 45.3 (10.3) | 48.6 (10.5) | 43.3 (9.2) | 45.9 (10.2) |
| Total energy intake (kilocalorie/day) | 2,074.0 (610.5) | 1,947.7 (622.6) | 2,024.2 (593.5) | 1,950.9 (591.4) | 1,973.9 (623.0) | 1,892.2 (602.4) | 2,154.8 (708.9) | 2,043.7 (646.1) |
| Alcohol consumption (gram/day) | 5.8 (0.9, 15.0) | 5.2 (0.9, 15.7) | 6.0 (0.9, 15.9) | 6.1 (1.0, 14.6) | 6.6 (1.0, 16.3) | 4.6 (0.9, 12.2) | 10.0 (2.7, 24.2) | 7.3 (2.0, 17.8) |
| Self-reported hypertension, % | 18.5 | 30.2 | 20.8 | 29.9 | 27.4 | 34.6 | 40.1 | 38.5 |
| Self-reported hypercholesterolemia, % | 28.3 | 37.8 | 28.6 | 38.7 | 34.5 | 39.9 | 46.3 | 43.9 |
| Family history of diabetes, % | 17.9 | 21.9 | 19.3 | 21.0 | 19.4 | 21.8 | 21.8 | 26.6 |
| **NHS** | | | | | | | | |
| Participants, number | 10,318 | 4,647 | 819 | 8,401 | 1,923 | 7,807 | 342 | 763 |
| Age in 1992 (year) | 58.5 (7.1) | 58.1 (7.0) | 57.7 (7.2) | 57.1 (6.9) | 55.2 (6.6) | 56.3 (6.7) | 55.8 (6.5) | 53.8 (6.1) |
| Ethnicity |  |  |  |  |  |  |  |  |
| White, % | 97.9 | 98.4 | 97.6 | 98.4 | 97.4 | 98.1 | 95.5 | 97.1 |
| African American, % | 0.8 | 1.0 | 1.4 | 1.1 | 1.9 | 1.3 | 1.7 | 2.2 |
| Asian, % | 0.2 | 0.2 | 0.5 | 0.2 | 0.2 | 0.2 | 0.5 | 0 |
| Other, % | 1.0 | 0.3 | 0.5 | 0.3 | 0.4 | 0.3 | 1.7 | 0.7 |
| Missing, % | 0 | 0 | 0 | 0 | 0.1 | 0 | 0.5 | 0 |
| Body mass index (kg/m²) | 23.1 (3.6) | 26.7 (4.9) | 25.4 (4.2) | 26 (4.1) | 26.5 (4.8) | 27.6 (4.7) | 26.3 (4.2) | 28.3 (5.4) |
| Waist circumference (centimeter) | 73.9 (8.6) | 80.4 (10.4) | 78.2 (9.4) | 79.4 (9.9) | 80.1 (11.5) | 82.1 (10.6) | 80.7 (11.2) | 82.3 (11.1) |
| Smoking status |  |  |  |  |  |  |  |  |
| Never smoker, % | 45.8 | 44.4 | 47.2 | 46.9 | 43.4 | 43.0 | 43.8 | 40.8 |
| Past smoker, % | 32.1 | 37.4 | 33.9 | 40.0 | 35.4 | 44.4 | 40.8 | 44.0 |
| Current smoker, % | 22.0 | 18.1 | 18.6 | 13.0 | 21.1 | 12.4 | 15.4 | 14.8 |
| Missing, % | 0.2 | 0.1 | 0.4 | 0.2 | 0.1 | 0.2 | 0 | 0.4 |
| Multivitamin use, % | 36.4 | 35.5 | 39.2 | 40.1 | 37.4 | 39.5 | 46.4 | 40.3 |
| Television watching (hour) |  |  |  |  |  |  |  |  |
| 0-1, % | 7.7 | 6.7 | 9.7 | 6.7 | 8.4 | 5.3 | 5.3 | 5.8 |
| 2-5, % | 24.6 | 22.5 | 29.6 | 23.7 | 22.3 | 22.8 | 28.1 | 23.2 |
| 6-10, % | 25.6 | 25.1 | 23.9 | 26.7 | 26.4 | 26.2 | 23.1 | 24.5 |
| 11-20, % | 26.1 | 26.9 | 21.7 | 26.7 | 24.5 | 27.2 | 23.0 | 25.7 |
| 21+, % | 15.0 | 16.7 | 12.9 | 14.5 | 17.4 | 17.2 | 18.6 | 19.9 |
| Missing, % | 1.1 | 2.1 | 2.2 | 1.7 | 1.0 | 1.3 | 1.9 | 0.8 |
| Physical activity (METs-hour/week) | 8.4 (3.2, 20.7) | 6.2 (2.3, 16.2) | 11.9 (4.7, 27.9) | 11.3 (4.5, 23.5) | 8.2 (2.9, 20.4) | 8.4 (3.3, 20.2) | 9.3 (3.8, 21.1) | 7.9 (2.7, 20.2) |
| Alternative healthy eating index | 44.9 (10.6) | 46.1 (10.3) | 47.3 (10.3) | 48.0 (10.4) | 45.1 (10.6) | 49.2 (10.5) | 46.4 (10.5) | 47.5 (11.2) |
| Total energy intake (kilocalorie/day) | 1808.6 (527.7) | 1747.8 (517.8) | 1777.3 (533.4) | 1758.2 (515.7) | 1741.9 (553.8) | 1727.8 (521.8) | 1697.3 (517.0) | 1738.9 (538.9) |
| Alcohol consumption (gram/day) | 2.0 (0, 9.5) | 1.8 (0, 6.9) | 1.8 (0, 7.6) | 1.8 (0, 7.5) | 1.9 (0, 7.8) | 1.8 (0, 6.0) | 1.5 (0, 4.7) | 2.0 (0, 6.9) |
| Self-reported hypertension, % | 22.1 | 33.2 | 29.5 | 31.3 | 34.5 | 35.5 | 27.4 | 38.4 |
| Self-reported hypercholesterolemia, % | 35.9 | 43.7 | 40.5 | 44.5 | 43.0 | 46.2 | 42.1 | 50.6 |
| Family history of diabetes, % | 25.7 | 28.9 | 27.9 | 29.3 | 30.7 | 30.6 | 32.1 | 31.4 |
| **NHSII** | | | | | | | | |
| Participants, number | 12,193 | 3,694 | 6,383 | 10,656 | 5,445 | 12,043 | 1,059 | 2,868 |
| Age in 1993 (year) | 38.1 (4.7) | 38.7 (4.6) | 37.9 (4.7) | 38.3 (4.6) | 37.9 (4.6) | 38.6 (4.6) | 37.9 (4.6) | 38.1 (4.6) |
| Ethnicity |  |  |  |  |  |  |  |  |
| White, % | 95.9 | 97.5 | 96.8 | 97.0 | 95.9 | 97.2 | 95.2 | 95.7 |
| African American, % | 1.1 | 1.3 | 1.4 | 1.5 | 2.2 | 1.7 | 2.9 | 2.7 |
| Asian, % | 0.3 | 0.3 | 0.5 | 0.4 | 0.5 | 0.3 | 1.0 | 0.7 |
| Other, % | 2.6 | 0.8 | 1.2 | 0.9 | 1.3 | 0.8 | 0.8 | 0.9 |
| Missing, % | 0.1 | 0.1 | 0.2 | 0.1 | 0.2 | 0.1 | 0.1 | 0 |
| Body mass index (kg/m²) | 21.3 (3.5) | 25.8 (5.5) | 24.2 (4.4) | 24.8 (4.6) | 24.4 (4.9) | 26.6 (5.1) | 25.5 (4.7) | 26.6 (5.4) |
| Waist circumference (centimeter) | 73.0 (9.6) | 82.7 (14.3) | 77.9 (11.8) | 80.0 (12.1) | 79.0 (12.7) | 84.8 (13.6) | 81.4 (13.5) | 85.0 (14.4) |
| Smoking status |  |  |  |  |  |  |  |  |
| Never smoker, % | 70.9 | 64.5 | 62.9 | 66.6 | 57.0 | 65.4 | 60.4 | 58.0 |
| Past smoker, % | 17.3 | 20.1 | 24.7 | 22.5 | 22.5 | 23.6 | 22.1 | 25.4 |
| Current smoker, % | 11.7 | 15.4 | 12.2 | 10.7 | 20.4 | 10.9 | 17.4 | 16.6 |
| Missing, % | 0.1 | 0.1 | 0.1 | 0.2 | 0.1 | 0.2 | 0.1 | 0 |
| Multivitamin use, % | 44.1 | 38.0 | 45.2 | 44.8 | 41.5 | 42.7 | 44.4 | 45.1 |
| Television watching (hour) |  |  |  |  |  |  |  |  |
| 0-1, % | 12.8 | 8.3 | 11.3 | 10.1 | 10.4 | 7.9 | 8.7 | 7.3 |
| 2-5, % | 33.4 | 30.8 | 32.9 | 32.8 | 30.9 | 28.8 | 30.7 | 30.4 |
| 6-10, % | 26.3 | 27.0 | 26.4 | 27.1 | 25.5 | 27.7 | 27.3 | 26.2 |
| 11-20, % | 17.2 | 20.1 | 17.0 | 18.7 | 19.0 | 21.8 | 19.7 | 19.8 |
| 21+, % | 5.4 | 7.4 | 5.3 | 5.7 | 7.0 | 8.0 | 6.2 | 8.6 |
| Missing, % | 5.0 | 6.4 | 7.2 | 5.7 | 7.2 | 5.8 | 7.4 | 7.6 |
| Physical activity (METs-hour/week) | 11.7 (4.5, 27.2) | 8.8 (3.5, 20.7) | 19.4 (7.8, 37.9) | 16.5 (7.2, 33.6) | 13.7 (5.2, 32.0) | 12.7 (5.1, 26.8) | 13.5 (4.9, 32.3) | 14.0 (4.9, 31.9) |
| Alternative healthy eating index | 42.0 (10.3) | 43.0 (10.2) | 46.7 (10.6) | 45.7 (10.4) | 42.9 (10.3) | 46.1 (10.5) | 43.1 (9.9) | 44.4 (10.5) |
| Total energy intake (kilocalorie/day) | 1,828.4 (537.2) | 1,777.3 (558.7) | 1,777.9 (548.4) | 1,775.4 (531.5) | 1,747.7 (578.0) | 1,779.7 (528.5) | 1,714.9 (543.4) | 1,754.8 (564.7) |
| Alcohol consumption (gram/day) | 0.9 (0, 3.4) | 0.9 (0, 2.9) | 0.9 (0, 3.6) | 0.9 (0, 3.5) | 1.1 (0, 4.7) | 0.9 (0, 2.9) | 1.1 (0, 3.9) | 0.9 (0, 3.5) |
| Self-reported hypertension, % | 3.8 | 9.5 | 7.1 | 8.1 | 8.5 | 10.4 | 9.0 | 11.7 |
| Self-reported hypercholesterolemia, % | 12.4 | 19.4 | 17.7 | 17.4 | 18.6 | 21.5 | 21.6 | 25.8 |
| Family history of diabetes, % | 13.2 | 17.7 | 16.2 | 17.5 | 18.1 | 18.5 | 18.7 | 20.2 |

Values are means (standard deviation) or medians (Q25, Q75) for continuous variables; percentages for categorical variables, and are standardized to the age distribution of the study population. Values of polytomous variables may not sum to 100% due to rounding. **Abbreviations**: CWLP, commercial weight loss program; FCP, select at least two strategies among fasting, CWLP, and pill; HPFS, Health Professionals Follow-up Study; kg/m^2^, kilogram per square meter; LCD, low-calorie diet; METs, metabolic equivalents of tasks; NHS, Nurses’ Health Study.
