# Supplementary material for: Weight loss strategies, weight change, and type 2 diabetes in US health professionals: A cohort study
Source: PLoS Med. 2022 Sep 27;19(9):e1004094. doi: 10.1371/journal.pmed.1004094 (PMC9514663; doi:10.1371/journal.pmed.1004094)
Supplement: S5 Table — (DOCX) [file pmed.1004094.s009.docx]

**S5 Table. Age-standardized characteristics of participants before weight loss in the weight change analyses.**

| **Characteristic** | **Reference** | **LCD** | **Exercise** | **LCD & Exercise** | **Fasting** | **CWLP** | **Pill** | **FCP** |
| --- | --- | --- | --- | --- | --- | --- | --- | --- |
| **HPFS** | | | | | | | | |
| Participants, number | 4,350 | 1,109 | 638 | 2,294 | 1,657 | 591 | 22 | 150 |
| Age in 1992 (year) | 53.7 (5.9) | 54.3 (5.8) | 52.7 (5.5) | 53.3 (5.6) | 52.8 (5.5) | 53.0 (5.5) | 52.9 (5.3) | 51.9 (5.4) |
| Ethnicity |  |  |  |  |  |  |  |  |
| White, % | 95.7 | 96.1 | 94.8 | 96.9 | 96.1 | 96.7 | 95.9 | 95.8 |
| African American, % | 1.7 | 2.6 | 2.0 | 1.8 | 2.3 | 2.5 | 4.1 | 1.1 |
| Asian, % | 2.0 | 0.9 | 2.1 | 0.6 | 0.9 | 0.3 | 0 | 0.6 |
| Other, % | 0.5 | 0.4 | 1.1 | 0.6 | 0.7 | 0.5 | 0 | 2.5 |
| Body mass index (kg/m²) | 24.0 (2.5) | 26.8 (3.2) | 25.5 (2.6) | 26.4 (2.9) | 26.6 (3.0) | 28.1 (3.3) | 28.3 (4.8) | 28.4 (3.3) |
| Waist circumference (centimeter) | 90.1 (7.4) | 97.9 (8.9) | 93.2 (7.9) | 96.2 (8.3) | 97.1 (8.8) | 101.6 (9.4) | 100.1 (13.7) | 101.7 (10.0) |
| Smoking status |  |  |  |  |  |  |  |  |
| Never smoker, % | 55.8 | 46.6 | 50.0 | 45.2 | 47.1 | 42.3 | 39.4 | 44.2 |
| Past smoker, % | 31.7 | 38.8 | 38.1 | 44.5 | 40.7 | 47.0 | 44.0 | 41.4 |
| Current smoker, % | 9.5 | 9.6 | 8.2 | 6.1 | 8.7 | 7.4 | 12.5 | 8.9 |
| Missing, % | 3.1 | 4.9 | 3.7 | 4.1 | 3.5 | 3.4 | 4.1 | 5.5 |
| Multivitamin use, % | 36.9 | 35.4 | 40.6 | 40.6 | 41.0 | 42.1 | 29.5 | 42.8 |
| Television watching (hour) |  |  |  |  |  |  |  |  |
| 0-1, % | 5.9 | 3.4 | 5.8 | 3.9 | 4.1 | 3.8 | 4.1 | 5.4 |
| 2-5, % | 29.5 | 24.9 | 30.8 | 27.8 | 27.1 | 25.4 | 28.3 | 25.9 |
| 6-10, % | 29.3 | 30.1 | 27.9 | 30.1 | 28.3 | 30.0 | 28.0 | 31.9 |
| 11-20, % | 27.8 | 31.1 | 29.8 | 29.7 | 29.8 | 29.3 | 35.3 | 23.2 |
| 21+, % | 7.2 | 10.3 | 5.3 | 8.3 | 10.4 | 10.5 | 4.4 | 13.6 |
| Missing, % | 0.3 | 0.2 | 0.3 | 0.1 | 0.3 | 1.0 | 0 | 0 |
| Physical activity (METs-hour/week) | 19.4 (7.7, 38.8) | 14.1 (5.3, 31.7) | 29.0 (12.9, 58.7) | 23.9 (11.1, 45.9) | 18.0 (6.9, 37.3) | 15.0 (5.3, 31.3) | 19.1 (5.1, 57.4) | 15.1 (4.5, 36.7) |
| Alternative healthy eating index | 44.6 (10.9) | 46.2 (10.6) | 46.7 (10.7) | 48.1 (10.6) | 44.7 (10.1) | 48.0 (10.6) | 42.7 (8.3) | 45.9 (10.9) |
| Total energy intake (kilocalorie/day) | 2092.5 (615.0) | 1953.4 (615.2) | 2029.3 (585.1) | 1960.6 (592.2) | 1974.9 (631.9) | 1905.9 (611.8) | 2090.7 (636.7) | 2059.7 (662.7) |
| Alcohol consumption (gram/day) | 5.5 (0.9, 14.6) | 4.6 (0, 14.0) | 5.8 (0, 15.8) | 6.4 (1.0, 14.8) | 6.3 (1.1, 16.1) | 4.5 (0.9, 12.1) | 12.9 (3.8, 25.8) | 5.8 (1.1, 14.8) |
| Self-reported hypertension, % | 14.6 | 26.8 | 18.7 | 25.7 | 23.5 | 30.3 | 43.4 | 35.0 |
| Self-reported hypercholesterolemia, % | 27.9 | 38.3 | 28.9 | 38.6 | 34.4 | 42.6 | 34.4 | 45.3 |
| Family history of diabetes, % | 17.7 | 21.5 | 18.7 | 21.1 | 18.8 | 22.1 | 16.3 | 28.7 |
| **NHS** | | | | | | | | |
| Participants, number | 7,613 | 3,517 | 617 | 6,791 | 1,676 | 6,600 | 303 | 707 |
| Age in 1992 (year) | 55.3 (5.3) | 55.2 (5.3) | 54.6 (5.4) | 54.6 (5.3) | 53.4 (5.0) | 54.3 (5.2) | 54.3 (5.3) | 52.8 (5.0) |
| Ethnicity |  |  |  |  |  |  |  |  |
| White, % | 97.8 | 98.3 | 97.2 | 98.4 | 97.4 | 98.1 | 95.8 | 96.8 |
| African American, % | 0.9 | 1.1 | 1.5 | 1.1 | 1.8 | 1.3 | 1.6 | 2.3 |
| Asian, % | 1.1 | 0.4 | 0.7 | 0.3 | 0.5 | 0.3 | 1.5 | 0.9 |
| Other, % | 0.3 | 0.2 | 0.6 | 0.2 | 0.3 | 0.3 | 1.1 | 0 |
| Body mass index (kg/m²) | 23.0 (3.6) | 26.7 (4.9) | 25.3 (4.1) | 25.9 (4.2) | 26.3 (4.8) | 27.6 (4.8) | 26.4 (4.3) | 28.2 (5.4) |
| Waist circumference (centimeter) | 73.1 (8.4) | 79.8 (10.4) | 77.6 (9.2) | 78.6 (9.8) | 79.3 (11.3) | 81.4 (10.6) | 79.3 (10.5) | 81.6 (11.6) |
| Smoking status |  |  |  |  |  |  |  |  |
| Never smoker, % | 45.7 | 43.6 | 46.1 | 46.1 | 40.9 | 42.4 | 45.0 | 41.1 |
| Past smoker, % | 31.1 | 37.3 | 33.3 | 39.7 | 36.0 | 44.3 | 39.1 | 41.3 |
| Current smoker, % | 23.1 | 19.0 | 20.1 | 14.1 | 22.9 | 13.2 | 16.0 | 17.1 |
| Missing, % | 0.1 | 0.1 | 0.5 | 0.2 | 0.2 | 0.2 | 0 | 0.4 |
| Multivitamin use, % | 35.4 | 34.2 | 39.5 | 39.1 | 37.0 | 38.3 | 46.6 | 42.1 |
| Television watching (hour) |  |  |  |  |  |  |  |  |
| 0-1, % | 8.5 | 7.4 | 10.5 | 7.4 | 8.9 | 6.0 | 5.4 | 6.9 |
| 2-5, % | 25.7 | 23.8 | 31.1 | 24.7 | 23.1 | 23.3 | 28.0 | 25.1 |
| 6-10, % | 26.8 | 25.9 | 24.9 | 27.9 | 27.9 | 27.3 | 22.4 | 25.5 |
| 11-20, % | 25.4 | 26.6 | 20.6 | 26.1 | 23.5 | 27.6 | 23.7 | 23.4 |
| 21+, % | 12.6 | 14.4 | 11.1 | 12.3 | 15.3 | 14.8 | 19.1 | 18.2 |
| Missing, % | 1.0 | 1.9 | 1.9 | 1.6 | 1.3 | 1.1 | 1.4 | 1.0 |
| Physical activity (METs-hour/week) | 8.1 (3.1, 20.2) | 5.9 (2.3, 15.9) | 12.2 (4.9, 27.9) | 10.9 (4.3, 22.7) | 8.1 (2.9, 20.2) | 8.4 (3.2, 20.0) | 7.9 (3.4, 20.4) | 7.9 (2.9, 20.1) |
| Alternative healthy eating index | 44.2 (10.5) | 45.4 (10.1) | 46.6 (10.1) | 47.3 (10.4) | 44.6 (10.5) | 48.5 (10.4) | 44.7 (9.3) | 46.8 (10.8) |
| Total energy intake (kilocalorie/day) | 1816.2 (530.3) | 1760.7 (519.2) | 1771.9 (524.9) | 1763.2 (517.5) | 1761.9 (562.9) | 1737.4 (527.5) | 1725.5 (525.2) | 1766.0 (551.8) |
| Alcohol consumption (gram/day) | 1.9 (0, 9.1) | 1.8 (0, 6.9) | 1.8 (0, 7.6) | 1.9 (0, 7.4) | 2.0 (0, 7.8) | 1.8 (0, 6.0) | 1.1 (0, 5.8) | 2.0 (0, 7.0) |
| Self-reported hypertension, % | 18.8 | 29.6 | 26.2 | 28.5 | 29.9 | 31.9 | 25.8 | 32.8 |
| Self-reported hypercholesterolemia, % | 33.0 | 40.8 | 37.6 | 41.4 | 40.1 | 43.8 | 39.6 | 46.0 |
| Family history of diabetes, % | 25.6 | 28.6 | 28.7 | 29.7 | 30.6 | 30.5 | 30.1 | 30.1 |
| **NHSII** | | | | | | | | |
| Participants, number | 12,201 | 3,700 | 6,403 | 10,688 | 5,463 | 12,078 | 1,063 | 2,879 |
| Age in 1993 (year) | 38.1 (4.7) | 38.7 (4.6) | 37.9 (4.7) | 38.3 (4.6) | 37.9 (4.6) | 38.6 (4.6) | 37.9 (4.6) | 38.1 (4.6) |
| Ethnicity |  |  |  |  |  |  |  |  |
| White, % | 95.9 | 97.5 | 96.8 | 97.0 | 95.9 | 97.2 | 95.1 | 95.7 |
| African American, % | 1.1 | 1.3 | 1.4 | 1.6 | 2.2 | 1.7 | 3.0 | 2.8 |
| Asian, % | 2.6 | 0.8 | 1.2 | 0.9 | 1.3 | 0.8 | 0.8 | 0.9 |
| Other, % | 0.4 | 0.3 | 0.7 | 0.5 | 0.6 | 0.4 | 1.1 | 0.7 |
| Body mass index (kg/m²) | 21.3 (3.4) | 25.8 (5.5) | 24.2 (4.4) | 24.8 (4.6) | 24.4 (4.9) | 26.6 (5.1) | 25.5 (4.7) | 26.6 (5.4) |
| Waist circumference (centimeter) | 73.0 (9.6) | 82.7 (14.3) | 77.9 (11.8) | 80.0 (12.1) | 79.0 (12.6) | 84.8 (13.6) | 81.5 (13.4) | 84.9 (14.3) |
| Smoking status |  |  |  |  |  |  |  |  |
| Never smoker, % | 70.9 | 64.6 | 62.8 | 66.6 | 57.1 | 65.3 | 60.4 | 57.9 |
| Past smoker, % | 17.3 | 20.0 | 24.8 | 22.5 | 22.4 | 23.6 | 22.0 | 25.3 |
| Current smoker, % | 11.7 | 15.4 | 12.2 | 10.7 | 20.5 | 10.9 | 17.6 | 16.8 |
| Missing, % | 0.1 | 0.1 | 0.1 | 0.2 | 0.1 | 0.2 | 0.1 | 0 |
| Multivitamin use, % | 44.1 | 38.0 | 45.2 | 44.8 | 41.6 | 42.8 | 44.4 | 45.0 |
| Television watching (hour) |  |  |  |  |  |  |  |  |
| 0-1, % | 12.8 | 8.4 | 11.3 | 10.1 | 10.4 | 7.9 | 8.7 | 7.3 |
| 2-5, % | 33.4 | 30.7 | 32.9 | 32.8 | 30.8 | 28.8 | 30.4 | 30.3 |
| 6-10, % | 26.3 | 27.1 | 26.3 | 27.0 | 25.5 | 27.7 | 27.3 | 26.1 |
| 11-20, % | 17.2 | 20.0 | 16.9 | 18.7 | 18.9 | 21.9 | 19.9 | 19.9 |
| 21+, % | 5.4 | 7.3 | 5.3 | 5.7 | 7.0 | 8.0 | 6.2 | 8.6 |
| Missing, % | 5.0 | 6.5 | 7.3 | 5.7 | 7.3 | 5.9 | 7.5 | 7.8 |
| Physical activity (METs-hour/week) | 11.7 (4.5, 27.2) | 8.8 (3.5, 20.7) | 19.3 (7.7, 37.8) | 16.6 (7.2, 33.7) | 13.7 (5.2, 31.9) | 12.7 (5.1, 26.9) | 13.4 (4.9, 32.3) | 14.0 (4.9, 32.1) |
| Alternative healthy eating index | 42.0 (10.3) | 43.0 (10.1) | 46.7 (10.6) | 45.6 (10.4) | 42.9 (10.3) | 46.1 (10.5) | 43.1 (9.9) | 44.4 (10.5) |
| Total energy intake (kilocalorie/day) | 1828.4 (537.9) | 1776.5 (559.0) | 1777.8 (548.4) | 1775.4 (531.8) | 1747.2 (578.3) | 1779.8 (528.4) | 1713.9 (543.6) | 1755.4 (565.6) |
| Alcohol consumption (gram/day) | 0.9 (0, 3.4) | 0.9 (0, 2.9) | 0.9 (0, 3.6) | 0.9 (0, 3.5) | 1.1 (0, 4.7) | 0.9 (0, 2.9) | 1.1 (0, 4.0) | 0.9 (0, 3.5) |
| Self-reported hypertension, % | 3.8 | 9.5 | 7.2 | 8.1 | 8.5 | 10.3 | 9.1 | 11.7 |
| Self-reported hypercholesterolemia, % | 12.4 | 19.4 | 17.8 | 17.4 | 18.7 | 21.5 | 21.9 | 25.7 |
| Family history of diabetes, % | 13.1 | 17.8 | 16.3 | 17.5 | 18.3 | 18.5 | 18.8 | 20.2 |

Values are means (standard deviation) or medians (Q25, Q75) for continuous variables; percentages for categorical variables, and are standardized to the age distribution of the study population. Values of polytomous variables may not sum to 100% due to rounding. **Abbreviations**: CWLP, commercial weight loss program; FCP, select at least two strategies among fasting, CWLP, and pill; HPFS, Health Professionals Follow-up Study; kg/m^2^, kilogram per square meter; LCD, low-calorie diet; METs, metabolic equivalents of tasks; NHS, Nurses’ Health Study.
