# Supplementary material for: Weight loss strategies, weight change, and type 2 diabetes in US health professionals: A cohort study
Source: PLoS Med. 2022 Sep 27;19(9):e1004094. doi: 10.1371/journal.pmed.1004094 (PMC9514663; doi:10.1371/journal.pmed.1004094)
Supplement: S6 Table — (DOCX) [file pmed.1004094.s010.docx]

**S6 Table. Comparison of characteristics before weight loss between participants who skipped the weight loss strategy question and those who did not.**

| **Characteristic** | **HPFS** | | **NHS** | | **NHSII** | |
| --- | --- | --- | --- | --- | --- | --- |
|  | **Skip** | **Not Skip** | **Skip** | **Not Skip** | **Skip** | **Not Skip** |
| Participants, number (%) | 17,118 (33.2) | 34,411 (66.8) | 42,188 (34.7) | 79,513 (65.3) | 26,504 (22.8) | 89,925 (77.2) |
| Age in 1992/1993 (year) | 61.7 (10.4) | 59.6 (9.6) | 59.5 (7.3) | 58.0 (7.1) | 38.4 (4.7) | 38.2 (4.7) |
| White race, % | 98.6 | 96.1 | 95.1 | 98.1 | 92.2 | 96.6 |
| Body mass index (kg/m²) | 25.2 (3.1) | 25.6 (3.1) | 25.2 (4.8) | 25.7 (4.7) | 24.0 (4.9) | 24.1 (4.8) |
| Waist circumference (centimeter) | 94.1 (8.7) | 95.4 (8.9) | 77.7 (10.7) | 78.9 (10.8) | 74.2 (10.8) | 78.2 (12.5) |
| Smoking status |  |  |  |  |  |  |
| Never smoker, % | 37.1 | 43.4 | 38.0 | 43.7 | 63.2 | 65.5 |
| Past smoker, % | 34.6 | 42.1 | 26.2 | 37.3 | 20.5 | 21.6 |
| Current smoker, % | 9.7 | 8.8 | 18.7 | 18.1 | 16.0 | 12.8 |
| Missing, % | 18.5 | 5.7 | 17.0 | 0.9 | 0.2 | 0.1 |
| Multivitamin use, % | 24.1 | 35.3 | 15.3 | 34.1 | 44.3 | 45.7 |
| Television watching (hour) |  |  |  |  |  |  |
| 0-1, % | 2.7 | 3.3 | 1.6 | 6.4 | 6.9 | 10.2 |
| 2-5, % | 17.3 | 21.3 | 5.9 | 23.5 | 20.8 | 31.7 |
| 6-10, % | 19.1 | 25.0 | 5.6 | 25.1 | 16.6 | 26.7 |
| 11-20, % | 18.9 | 26.3 | 5.3 | 26.2 | 11.0 | 18.8 |
| 21+, % | 7.8 | 10.7 | 3.4 | 17.2 | 3.9 | 6.4 |
| Missing, % | 34.2 | 13.4 | 78.1 | 1.6 | 40.9 | 6.2 |
| Physical activity (METs-hour/week) | 17.5 (6.5, 37.4) | 18.8 (7.5, 38.7) | 7.7 (2.9, 20.1) | 8.9 (3.2, 20.7) | 13.7 (5.0, 32.1) | 13.6 (5.3, 29.9) |
| Alternative healthy eating index | 47.1 (10.9) | 47.1 (10.9) | 46.9 (10.6) | 47.0 (10.7) | 43.8 (10.4) | 44.0 (10.5) |
| Total energy intake (kilocalorie/day) | 1972.8 (627.6) | 1992.2 (615.7) | 1756.1 (530.0) | 1767.1 (525.3) | 1780.8 (564.2) | 1790.9 (545.8) |
| Alcohol consumption (gram/day) | 5.3 (0, 14.3) | 6.0 (0.9, 15.3) | 1.5 (0, 7.9) | 1.8 (0, 7.6) | 0.9 (0, 3.3) | 0.9 (0, 3.5) |
| Self-reported hypertension, % | 30.9 | 30.9 | 26.0 | 32.8 | 8.5 | 8.0 |
| Self-reported hypercholesterolemia, % | 32.5 | 37.9 | 29.2 | 45.3 | 16.0 | 18.1 |
| Family history of diabetes, % | 11.0 | 21.6 | 17.0 | 30.3 | 17.2 | 16.9 |

Values are means (standard deviation) or medians (Q25, Q75) for continuous variables; percentages for categorical variables, and are standardized to the age distribution of the study population. Values of polytomous variables may not sum to 100% due to rounding. **Abbreviations**: HPFS, Health Professionals Follow-up Study; NHS, Nurses’ Health Study; METs, metabolic equivalent tasks.
