# Supplementary material for: Weight loss strategies, weight change, and type 2 diabetes in US health professionals: A cohort study
Source: PLoS Med. 2022 Sep 27;19(9):e1004094. doi: 10.1371/journal.pmed.1004094 (PMC9514663; doi:10.1371/journal.pmed.1004094)
Supplement: S8 Table — (DOCX) [file pmed.1004094.s012.docx]

**S8 Table. Proportions (95% CIs) of the association between weight loss strategies and type 2 diabetes mediated by time-varying body mass index.**

|  | **LCD** | **Exercise** | **LCD & Exercise** | **Fasting** | **CWLP** | **Pill** | **FCP** |
| --- | --- | --- | --- | --- | --- | --- | --- |
| **Proportions** | 27.9% | 15.6% | 23.3% | 18.9% | 42.2% | 27.7% | 46.8% |
| **95% CI** | 21.1%, 36.0% | 7.7%, 29.0% | 16.3%, 32.2% | 14.1%, 24.8% | 33.9%, 51.0% | 23.4%, 32.6% | 37.7%, 56.1% |
| ***P* value** | <0.001 | <0.001 | <0.001 | <0.001 | <0.001 | <0.001 | <0.001 |

The multivariable model was adjusted for cohort (Health Professionals Follow-up Study, Nurses’ Health Study, or Nurses’ Health Study II), age (in month, continuous), ethnicity (white, African American, Asian, or other), baseline body weight (in kilogram, continuous), baseline waist circumference (in centimeter, continuous), physical activity (in quintiles), television watching (0-1, 2-5, 6-10, 11-20, or >20 hour/week), smoking status (never, past, or current smokers), alcohol intake (0, <5.0, 5.0-9.9, 10.0-14.9, 15.0-29.9, or >30.0 gram/day), hypertension (yes or no), hypercholesterolemia (yes or no), family history of diabetes (yes or no), multivitamin use (yes or no), Alternative Healthy Eating Index score (in quintiles), and total energy intake (in quintiles) before weight loss. The mediator was the biennially-updated body mass index from 1992/1993 to 2016/2017. **Abbreviations**: CI, confidence interval; CWLP, commercial weight loss program; FCP, select at least two strategies among fasting, CWLP, and pill; LCD, low-calorie diet.
