# Supplementary material for: Weight loss strategies, weight change, and type 2 diabetes in US health professionals: A cohort study
Source: PLoS Med. 2022 Sep 27;19(9):e1004094. doi: 10.1371/journal.pmed.1004094 (PMC9514663; doi:10.1371/journal.pmed.1004094)
Supplement: S11 Table — (DOCX) [file pmed.1004094.s015.docx]

**S11 Table. Weight loss strategies and weight change percentages (maximum body mass index before 1992/1993).**

| **Weight Loss Strategies** | **Number of Participants**  **at Baseline** | **Adjusted for Age** | | | **Adjusted for Multiple Variables** | | |
| --- | --- | --- | --- | --- | --- | --- | --- |
|  |  | **Difference of Baseline Weight (kg)** | **Difference of Weight Change Percentage (%)** | | **Difference of Baseline Weight (kg)** | **Difference of Weight Change Percentage (%)** | |
|  |  |  | **Four Years since Baseline** | **Ten Years since Baseline** |  | **Four Years since Baseline** | **Ten Years since Baseline** |
| **Overall Population** | | | | | | | |
| Reference | 24,164 | 0 (Reference) | 0 (Reference) | 0 (Reference) | 0 (Reference) | 0 (Reference) | 0 (Reference) |
| LCD | 8,326 | 10.6 (10.2, 11.0) | 1.0 (0.8, 1.2) | 1.9 (1.6, 2.2) | 9.9 (9.6, 10.2) | 1.1 (0.9, 1.3) | 2.0 (1.7, 2.4) |
| Exercise | 7,658 | 7.3 (7.0, 7.6) | -0.7 (-0.9, -0.5) | 1.6 (1.3, 1.9) | 7.5 (7.2, 7.8) | -0.6 (-0.8, -0.4) | 1.7 (1.4, 2.0) |
| LCD & Exercise | 19,773 | 8.4 (8.2, 8.6) | 0.7 (0.6, 0.8) | 2.3 (2.1, 2.5) | 8.7 (8.5, 8.9) | 0.7 (0.6, 0.9) | 2.4 (2.2, 2.6) |
| Fasting | 8,796 | 9.3 (9.0, 9.7) | 0.7 (0.5, 0.9) | 2.2 (1.9, 2.5) | 8.0 (7.7, 8.3) | 0.9 (0.7, 1.1) | 2.4 (2.1, 2.7) |
| CWLP | 19,269 | 12.1 (11.8, 12.3) | 2.1 (1.9, 2.3) | 5.7 (5.5, 6.0) | 12.9 (12.7, 13.1) | 2.0 (1.8, 2.2) | 5.7 (5.5, 6.0) |
| Pill | 1,388 | 9.1 (8.4, 9.9) | 1.8 (1.2, 2.3) | 5.6 (4.9, 6.4) | 10.2 (9.6, 10.9) | 1.7 (1.1, 2.2) | 5.4 (4.6, 6.1) |
| FCP | 3,736 | 13.1 (12.6, 13.6) | 3.7 (3.3, 4.1) | 6.6 (6.1, 7.2) | 13.3 (12.9, 13.8) | 3.4 (3.0, 3.9) | 6.6 (6.0, 7.1) |
| **Baseline Body Mass Index < 25 kg/m^2^** | | | | | | | |
| Reference | 18,540 | 0 (Reference) | 0 (Reference) | 0 (Reference) | 0 (Reference) | 0 (Reference) | 0 (Reference) |
| LCD | 2,919 | 2.7 (2.4, 3.0) | -0.2 (-0.4, 0.1) | 1.2 (0.9, 1.6) | 3.2 (3.0, 3.3) | 0.6 (0.4, 0.9) | 2.0 (1.7, 2.4) |
| Exercise | 4,024 | 3.5 (3.3, 3.8) | -1.3 (-1.5, -1.1) | 0.5 (0.2, 0.9) | 3.3 (3.1, 3.4) | -0.6 (-0.8, -0.4) | 1.3 (0.9, 1.6) |
| LCD & Exercise | 7,943 | 3.2 (3.0, 3.4) | -0.1 (-0.3, 0.0) | 1.6 (1.3, 1.8) | 3.5 (3.4, 3.6) | 0.7 (0.5, 0.8) | 2.4 (2.2, 2.7) |
| Fasting | 3,984 | 3.2 (2.9, 3.5) | 0.0 (-0.3, 0.2) | 1.6 (1.2, 1.9) | 2.8 (2.6, 2.9) | 0.5 (0.3, 0.8) | 2.2 (1.9, 2.6) |
| CWLP | 4,468 | 3.6 (3.4, 3.8) | -0.5 (-0.7, -0.3) | 3.6 (3.3, 4.0) | 4.9 (4.7, 5.0) | 0.5 (0.3, 0.7) | 4.7 (4.3, 5.0) |
| Pill | 472 | 2.9 (2.2, 3.5) | -0.3 (-0.9, 0.3) | 4.1 (3.0, 5.2) | 3.8 (3.4, 4.1) | 0.4 (-0.2, 1.1) | 4.8 (3.6, 5.9) |
| FCP | 957 | 3.3 (2.8, 3.7) | 0.6 (0.1, 1.0) | 3.7 (2.9, 4.4) | 3.9 (3.6, 4.2) | 1.2 (0.8, 1.7) | 4.5 (3.7, 5.2) |
| **Baseline Body Mass Index 25 to < 30 kg/m^2^** | | | | | | | |
| Reference | 4,250 | 0 (Reference) | 0 (Reference) | 0 (Reference) | 0 (Reference) | 0 (Reference) | 0 (Reference) |
| LCD | 3,080 | 1.4 (0.9, 1.9) | -1.2 (-1.6, -0.9) | 0.2 (-0.4, 0.7) | 1.7 (1.4, 1.9) | -0.7 (-1.0, -0.3) | 0.7 (0.1, 1.2) |
| Exercise | 2,448 | 1.1 (0.5, 1.6) | -2.2 (-2.6, -1.8) | 1.1 (0.5, 1.7) | 1.6 (1.3, 1.8) | -2.1 (-2.5, -1.7) | 1.2 (0.6, 1.8) |
| LCD & Exercise | 7,743 | 0.6 (0.2, 1.0) | -1.2 (-1.5, -0.9) | 0.9 (0.5, 1.4) | 1.6 (1.4, 1.7) | -1.0 (-1.2, -0.7) | 1.2 (0.7, 1.6) |
| Fasting | 3,111 | 3.2 (2.6, 3.7) | -1.2 (-1.5, -0.8) | 0.6 (0.1, 1.2) | 1.5 (1.2, 1.7) | -0.6 (-1.0, -0.3) | 1.0 (0.5, 1.6) |
| CWLP | 7,741 | -0.7 (-1.0, -0.3) | -0.8 (-1.1, -0.5) | 3.8 (3.4, 4.3) | 1.8 (1.6, 2.0) | -0.6 (-0.9, -0.4) | 4.0 (3.5, 4.4) |
| Pill | 510 | -1.7 (-2.5, -0.9) | -0.5 (-1.3, 0.4) | 4.1 (2.9, 5.3) | 1.2 (0.7, 1.7) | -0.7 (-1.5, 0.1) | 3.7 (2.5, 5.0) |
| FCP | 1,307 | 0.1 (-0.5, 0.7) | 0.1 (-0.4, 0.7) | 4.2 (3.4, 5.0) | 1.7 (1.3, 2.0) | 0.0 (-0.5, 0.6) | 4.3 (3.4, 5.1) |

**S11 Table. Weight loss strategies and weight change percentages (maximum body mass index before 1992/1993), continued.**

| **Weight Loss Strategies** | **Number of Participants**  **at Baseline** | **Adjusted for Age** | | | **Adjusted for Multiple Variables** | | |
| --- | --- | --- | --- | --- | --- | --- | --- |
|  |  | **Difference of Baseline Weight (kg)** | **Difference of Weight Change Percentage (%)** | | **Difference of Baseline Weight (kg)** | **Difference of Weight Change Percentage (%)** | |
|  |  |  | **Four Years since Baseline** | **Ten Years since Baseline** |  | **Four Years since Baseline** | **Ten Years since Baseline** |
| **Baseline Body Mass Index≥ 30 kg/m^2^** | | | | | | | |
| Reference | 1,374 | 0 (Reference) | 0 (Reference) | 0 (Reference) | 0 (Reference) | 0 (Reference) | 0 (Reference) |
| LCD | 2,327 | 2.6 (1.5, 3.6) | -1.4 (-2.0, -0.7) | -0.6 (-1.6, 0.4) | 1.8 (1.0, 2.6) | -0.6 (-1.2, 0.1) | 0.0 (-1.0, 1.0) |
| Exercise | 1,186 | 0.4 (-0.8, 1.6) | -3.0 (-3.8, -2.1) | -0.3 (-1.5, 0.8) | 1.0 (0.0, 1.9) | -2.9 (-3.7, -2.1) | -0.2 (-1.4, 1.0) |
| LCD & Exercise | 4,087 | 0.0 (-1.0, 0.9) | -1.6 (-2.2, -1.0) | -0.5 (-1.4, 0.4) | 0.4 (-0.3, 1.1) | -1.8 (-2.3, -1.2) | -0.3 (-1.2, 0.6) |
| Fasting | 1,701 | 2.7 (1.6, 3.8) | -1.4 (-2.1, -0.6) | 0.3 (-0.8, 1.4) | 1.5 (0.6, 2.3) | -0.8 (-1.5, -0.1) | 0.8 (-0.2, 1.9) |
| CWLP | 7,060 | -0.2 (-1.1, 0.7) | 0.1 (-0.5, 0.7) | 3.2 (2.3, 4.0) | 0.6 (-0.1, 1.3) | 0.1 (-0.4, 0.7) | 3.3 (2.4, 4.1) |
| Pill | 406 | -2.3 (-3.9, -0.6) | 0.0 (-1.3, 1.4) | 3.2 (1.4, 5.0) | -1.1 (-2.4, 0.3) | -0.3 (-1.6, 1.0) | 2.5 (0.8, 4.3) |
| FCP | 1,472 | 0.2 (-0.9, 1.4) | 2.5 (1.6, 3.4) | 5.0 (3.8, 6.3) | 1.0 (0.0, 1.9) | 2.4 (1.5, 3.2) | 5.2 (3.9, 6.4) |

Least squares means (95% conference intervals) for baseline body weight and weight change percentage since baseline were calculated using generalized linear model and generalized estimating equation, respectively. For weight change percentage, the multivariable model was adjusted for cohort (Health Professionals Follow-up Study, Nurses’ Health Study, or Nurses’ Health Study II), age (in month, continuous), ethnicity (white, African American, Asian, or other), baseline body weight (in kilogram, continuous), baseline waist circumference (in centimeter, continuous), physical activity (in quintiles), television watching (0-1, 2-5, 6-10, 11-20, or >20 hour/week), smoking status (never, past, or current smokers), alcohol intake (0, <5.0, 5.0-9.9, 10.0-14.9, 15.0-29.9, or >30.0 gram/day), hypertension (yes or no), hypercholesterolemia (yes or no), family history of diabetes (yes or no), multivitamin use (yes or no), Alternative Healthy Eating Index score (in quintiles), and total energy intake (in quintiles) before weight loss. For baseline body weight, all abovementioned covariates were adjusted for except that body weight and waist circumference were replaced with height (in meter, continuous). **Abbreviations**: CWLP, commercial weight loss program; FCP, select at least two strategies among fasting, CWLP, and pill; LCD, low-calorie diet; kg, kilogram; kg/m^2^, kilogram per square meter. 1 kg = 2.2 lbs.
