# Supplementary material for: Weight loss strategies, weight change, and type 2 diabetes in US health professionals: A cohort study
Source: PLoS Med. 2022 Sep 27;19(9):e1004094. doi: 10.1371/journal.pmed.1004094 (PMC9514663; doi:10.1371/journal.pmed.1004094)
Supplement: S13 Table — (DOCX) [file pmed.1004094.s017.docx]

**S13 Table. Baseline weight and weight change percentage since baseline across different weight loss strategies stratified by baseline abdominal obesity status.**

| **Weight Loss Strategies** | **Number of Participants**  **at Baseline** | **Adjusted for Age** | | | | | **Adjusted for Multiple Variables** | | | | |
| --- | --- | --- | --- | --- | --- | --- | --- | --- | --- | --- | --- |
|  |  | **Difference of Baseline Weight (kg)** | | **Difference of Weight Change Percentage (%)** | | | **Difference of Baseline Weight (kg)** | | **Difference of Weight Change Percentage (%)** | | |
|  |  |  |  | **Four Years since Baseline** | | **Ten Years since Baseline** |  |  | **Four Years since Baseline** | **Ten Years since Baseline** | |
| **Without Abdominal Obesity** | | | | | | | | | | | |
| Reference | 22,205 | 0 (Reference) | 0 (Reference) | | 0 (Reference) | | 0 (Reference) | 0 (Reference) | | | 0 (Reference) |
| LCD | 5,850 | 6.3 (5.9, 6.6) | 0.9 (0.6, 1.1) | | 2.1 (1.7, 2.4) | | 6.6 (6.3, 6.8) | 1.1 (0.8, 1.3) | | | 2.3 (1.9, 2.7) |
| Exercise | 6,204 | 5.6 (5.3, 5.9) | -0.9 (-1.1, -0.6) | | 1.7 (1.4, 2.0) | | 5.9 (5.7, 6.1) | -0.6 (-0.8, -0.4) | | | 1.9 (1.5, 2.2) |
| LCD & Exercise | 15,271 | 6.0 (5.8, 6.2) | 0.7 (0.5, 0.8) | | 2.4 (2.2, 2.6) | | 6.6 (6.5, 6.8) | 0.9 (0.7, 1.1) | | | 2.6 (2.4, 2.9) |
| Fasting | 6,760 | 6.4 (6.1, 6.7) | 0.6 (0.4, 0.8) | | 2.3 (1.9, 2.6) | | 5.6 (5.4, 5.9) | 0.9 (0.6, 1.2) | | | 2.6 (2.3, 3.0) |
| CWLP | 12,586 | 7.7 (7.4, 7.9) | 1.9 (1.7, 2.1) | | 5.9 (5.7, 6.2) | | 9.4 (9.2, 9.6) | 2.1 (1.9, 2.4) | | | 6.1 (5.8, 6.4) |
| Pill | 993 | 5.7 (5.1, 6.3) | 1.6 (1.0, 2.1) | | 5.5 (4.6, 6.3) | | 7.6 (7.1, 8.1) | 1.8 (1.1, 2.5) | | | 5.7 (4.7, 6.6) |
| FCP | 2,315 | 7.7 (7.3, 8.2) | 3.1 (2.6, 3.6) | | 6.7 (6.0, 7.3) | | 8.9 (8.5, 9.3) | 3.3 (2.8, 3.8) | | | 6.9 (6.3, 7.5) |
| **Abdominal Obesity** | | | | | | | | | | | |
| Reference | 1,959 | 0 (Reference) | 0 (Reference) | | 0 (Reference) | | 0 (Reference) | 0 (Reference) | | | 0 (Reference) |
| LCD | 2,476 | 6.5 (5.6, 7.5) | -0.5 (-1.0, 0.0) | | -0.5 (-1.4, 0.3) | | 6.3 (5.1, 7.4) | 0.1 (-0.5, 0.8) | | | 0.4 (-0.6, 1.4) |
| Exercise | 1,454 | 3.3 (2.2, 4.5) | -1.6 (-2.2, -1.0) | | -0.1 (-1.1, 0.8) | | 4.4 (3.2, 5.6) | -1.8 (-2.5, -1.1) | | | 0.3 (-0.8, 1.4) |
| LCD & Exercise | 4,502 | 3.8 (2.9, 4.6) | -1.0 (-1.4, -0.5) | | 0.1 (-0.6, 0.9) | | 4.4 (3.5, 5.2) | -0.7 (-1.2, -0.2) | | | 0.7 (-0.1, 1.6) |
| Fasting | 2,036 | 6.4 (5.3, 7.4) | -0.6 (-1.2, 0.0) | | 0.3 (-0.6, 1.2) | | 5.4 (4.4, 6.4) | -0.1 (-0.7, 0.6) | | | 0.8 (-0.2, 1.8) |
| CWLP | 6,683 | 5.3 (4.5, 6.2) | 0.5 (0.1, 1.0) | | 3.4 (2.7, 4.1) | | 6.4 (5.4, 7.4) | 0.8 (0.3, 1.2) | | | 3.8 (3.1, 4.6) |
| Pill | 395 | 3.6 (1.9, 5.3) | 1.4 (0.3, 2.5) | | 2.5 (0.9, 4.1) | | 4.9 (2.8, 7.0) | 0.6 (-0.9, 2.2) | | | 3.0 (1.2, 4.8) |
| FCP | 1,421 | 6.4 (5.2, 7.5) | 2.4 (1.7, 3.2) | | 4.8 (3.7, 5.8) | | 7.4 (6.3, 8.5) | 2.6 (1.7, 3.4) | | | 4.9 (3.8, 6.0) |

Least squares means (95% conference intervals) for baseline body weight and weight change percentage were calculated using generalized linear model and generalized estimating equation, respectively, with adjustment for cohort (Health Professionals Follow-up Study, Nurses’ Health Study, or Nurses’ Health Study II), age (in month, continuous), ethnicity (white, African American, Asian, or other ), baseline body weight (in kilogram, continuous), baseline waist circumference (in centimeter, continuous), physical activity (in quintiles), television watching (0-1, 2-5, 6-10, 11-20, or >20 hour/week), smoking status (never, past, or current smokers), alcohol intake (0, <5.0, 5.0-9.9, 10.0-14.9, 15.0-29.9, or >30.0 gram/day), hypertension (yes or no), hypercholesterolemia (yes or no), family history of diabetes (yes or no), multivitamin use (yes or no), Alternative Healthy Eating Index score (in quintiles), and total energy intake (in quintiles) before weight loss. For baseline body weight, all abovementioned covariates were adjusted for except that body weight and waist circumference were replaced with height (in meter, continuous).*P* for interaction for overall and individual weight loss strategies were less than 0.001. **Abbreviations**: CWLP, commercial weight loss program; FCP, select at least two strategies among fasting, CWLP, and pill; LCD, low-calorie diet.
