# Supplementary material for: Weight loss strategies, weight change, and type 2 diabetes in US health professionals: A cohort study
Source: PLoS Med. 2022 Sep 27;19(9):e1004094. doi: 10.1371/journal.pmed.1004094 (PMC9514663; doi:10.1371/journal.pmed.1004094)
Supplement: S14 Table — (DOCX) [file pmed.1004094.s018.docx]

**S14 Table. The hazard ratio of type 2 diabetes reduced by each unit increment of baseline waist circumference.**

|  | **Reference** | **LCD** | **Exercise** | **LCD & Exercise** | **Fasting** | **CWLP** | **Pill** | **FCP** |
| --- | --- | --- | --- | --- | --- | --- | --- | --- |
| **Hazard Ratio** | 1.000 | 0.979 | 0.986 | 0.983 | 0.984 | 0.976 | 0.977 | 0.971 |
| **95% CI** | - | 0.974, 0.984 | 0.979, 0.992 | 0.978, 0.988 | 0.978, 0.989 | 0.972, 0.981 | 0.966, 0.987 | 0.964, 0.978 |
| ***P* value** | - | <0.001 | <0.001 | <0.001 | <0.001 | <0.001 | <0.001 | <0.001 |

The multivariable model was adjusted for cohort (Health Professionals Follow-up Study, Nurses’ Health Study, or Nurses’ Health Study II), age (in month, continuous), ethnicity (white, African American, Asian, or other), baseline body mass index (in kg/m^2^, continuous), baseline waist circumference (in centimeter, continuous), physical activity (in quintiles), television watching (0-1, 2-5, 6-10, 11-20, or >20 hour/week), smoking status (never, past, or current smokers), alcohol intake (0, <5.0, 5.0-9.9, 10.0-14.9, 15.0-29.9, or >30.0 gram/day), hypertension (yes or no), hypercholesterolemia (yes or no), family history of diabetes (yes or no), multivitamin use (yes or no), Alternative Healthy Eating Index score (in quintiles), and total energy intake (in quintiles) before weight loss. **Abbreviations**: CI, confidence interval; CWLP, commercial weight loss program; FCP, select at least two strategies among fasting, CWLP, and pill; kg/m^2^, kilogram per square meter; LCD, low-calorie diet.
