# Supplementary material for: Weight loss strategies, weight change, and type 2 diabetes in US health professionals: A cohort study
Source: PLoS Med. 2022 Sep 27;19(9):e1004094. doi: 10.1371/journal.pmed.1004094 (PMC9514663; doi:10.1371/journal.pmed.1004094)
Supplement: S1 Fig — (PDF) [file pmed.1004094.s020.pdf]

S1 Fig. Flow chart of participants.

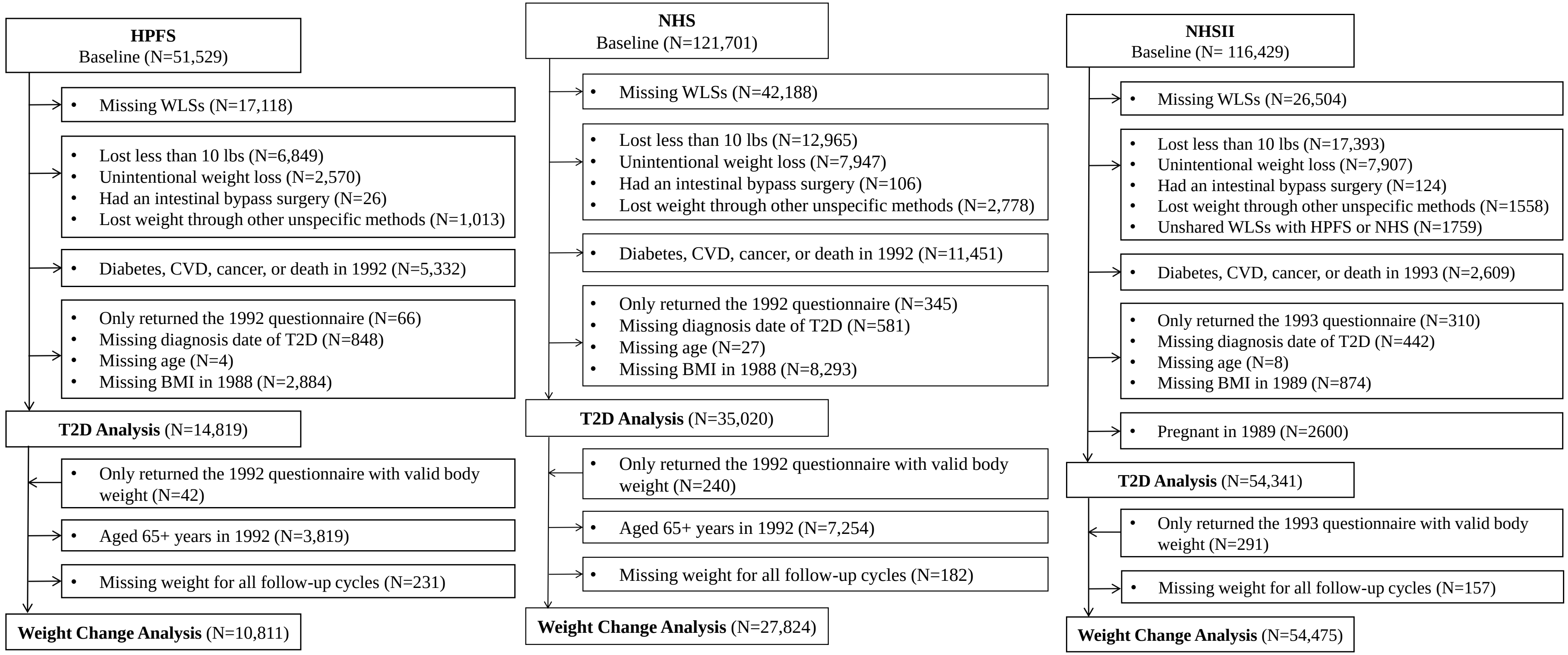

Abbreviations: BMI, body mass index; CVD, cardiovascular disease; HPFS, Health Professionals Follow-up Study; NHS, Nurses' Health Study; T2D, type 2 diabetes; WLS, weight loss strategy.
