# Supplementary material for: Weight loss strategies, weight change, and type 2 diabetes in US health professionals: A cohort study
Source: PLoS Med. 2022 Sep 27;19(9):e1004094. doi: 10.1371/journal.pmed.1004094 (PMC9514663; doi:10.1371/journal.pmed.1004094)
Supplement: S5 Fig — (PDF) [file pmed.1004094.s024.pdf]

S5 Fig. Pooled hazard ratios for association between weight loss strategies and the incidence of type 2 diabetes in participants < 65 years old.

Baseline BMI	Weight Loss Strategies	Cases/ Person-year	Hazard Ratio (95% Confidence Interval) Adjusted for Age	Adjusted for Multiple Covariates
Overall	Reference	1,189/524,805	1.00 (Reference)	1.00 (Reference)
	LCD	1,107/169,893	2.78 (2.56-3.02)	1.45 (1.33-1.57)
	Exercise	559/164,532	1.71 (1.54-1.89)	1.20 (1.08-1.33)
	LCD & Exercise	1,979/418,232	2.12 (1.97-2.27)	1.33 (1.23-1.43)
	Fasting	913/182,464	2.34 (2.15-2.55)	1.41 (1.29-1.54)
	CWLP	2,790/398,656	3.19 (2.98-3.42)	1.52 (1.41-1.64)
	Pill	180/28,729	3.08 (2.63-3.60)	1.67 (1.42-1.96)
	FCP	572/76,018	3.69 (3.34-4.08)	1.56 (1.41-1.74)
< 25 kg/m^2	Reference	515/444,698	1.00 (Reference)	1.00 (Reference)
	LCD	211/86,211	2.08 (1.77-2.44)	1.40 (1.18-1.65)
	Exercise	165/113,287	1.49 (1.24-1.78)	1.13 (0.94-1.36)
	LCD & Exercise	505/240,349	1.89 (1.67-2.14)	1.35 (1.18-1.53)
	Fasting	226/110,901	1.97 (1.68-2.31)	1.36 (1.16-1.60)
	CWLP	473/177,130	2.44 (2.15-2.78)	1.44 (1.25-1.65)
	Pill	42/16,228	2.57 (1.87-3.53)	1.57 (1.14-2.16)
	FCP	95/34,716	2.79 (2.24-3.48)	1.58 (1.25-1.98)
25 to < 30 kg/m^2	Reference	380/64,620	1.00 (Reference)	1.00 (Reference)
	LCD	409/55,326	1.25 (1.08-1.44)	1.15 (1.00-1.33)
	Exercise	198/37,448	0.93 (0.78-1.10)	0.91 (0.77-1.09)
	LCD & Exercise	771/130,274	1.01 (0.89-1.14)	0.98 (0.86-1.11)
	Fasting	348/51,184	1.20 (1.04-1.39)	1.10 (0.95-1.28)
	CWLP	1,020/142,461	1.21 (1.07-1.37)	1.15 (1.01-1.30)
	Pill	74/8,444	1.53 (1.19-1.98)	1.40 (1.09-1.81)
	FCP	215/25,140	1.47 (1.24-1.75)	1.29 (1.09-1.54)
≥ 30 kg/m^2	Reference	294/15,486	1.00 (Reference)	1.00 (Reference)
	LCD	487/28,356	0.93 (0.80-1.07)	0.86 (0.74-0.99)
	Exercise	196/13,798	0.79 (0.66-0.95)	0.79 (0.66-0.95)
	LCD & Exercise	703/47,610	0.81 (0.70-0.93)	0.78 (0.68-0.90)
	Fasting	339/20,380	0.90 (0.77-1.05)	0.86 (0.73-1.01)
	CWLP	1,297/79,065	0.90 (0.79-1.02)	0.86 (0.75-0.98)
	Pill	64/4,057	0.87 (0.66-1.14)	0.84 (0.64-1.11)
	FCP	262/16,162	0.90 (0.76-1.07)	0.82 (0.69-0.97)

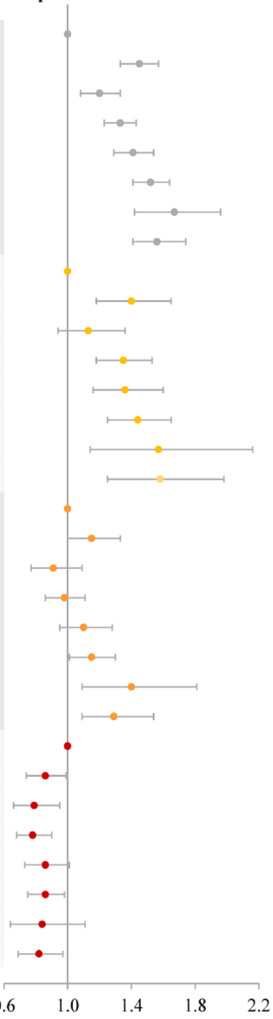

Hazard ratios and 95% confidence intervals were calculated using Cox proportional-hazards model. Multivariable model was adjusted for cohort (Health Professionals Follow-up Study, Nurses' Health Study, or Nurses' Health Study II), age (in months, continuous), ethnicity (white, African American, Asian, or other), baseline body mass index (in kg/m^2, continuous), baseline waist circumference (in cm, continuous), physical activity (in quintiles), television watching (0-1, 2-5, 6-10, 11-20, or >20 hour/week), smoking status (never, past, or current smokers), alcohol intake (0, <5.0, 5.0-9.9, 10.0-14.9, 15.0-29.9, or >30.0 gram/day), hypertension (yes or no), hypercholesterolemia (yes or no), family history of diabetes (yes or no), multivitamin use (yes or no), Alternative Healthy Eating Index score (in quintiles), and total energy intake (in quintiles) before weight loss. **Abbreviations:** BMI, body mass index; CWLP, commercial weight loss program; FCP, select at least two strategies among fasting, CWLP, and pill; LCD, low-calorie diet; kg/m^2, kilogram per square meter.
