# Supplementary material for: Weight loss strategies, weight change, and type 2 diabetes in US health professionals: A cohort study
Source: PLoS Med. 2022 Sep 27;19(9):e1004094. doi: 10.1371/journal.pmed.1004094 (PMC9514663; doi:10.1371/journal.pmed.1004094)
Supplement: S6 Fig — (PDF) [file pmed.1004094.s025.pdf]

S6 Fig. Pooled hazard ratios for association between weight loss strategies and the incidence of type 2 diabetes (participants who were diagnosed type 2 diabetes in the first 4-year follow-up were excluded from the analysis).

Baseline BMI	Weight Loss Strategies	Cases/ Person-year	Hazard Ratio (95% Confidence Interval) Adjusted for Age	Adjusted for Multiple Covariates
Overall	Reference	1,303/600,676	1.00 (Reference)	1.00 (Reference)
	LCD	1,070/194,892	2.47 (2.28-2.68)	1.35 (1.24-1.47)
	Exercise	533/170,097	1.59 (1.44-1.77)	1.15 (1.03-1.27)
	LCD & Exercise	1,936/453,710	1.98 (1.85-2.13)	1.29 (1.20-1.38)
	Fasting	860/191,560	2.16 (1.98-2.36)	1.36 (1.24-1.48)
	CWLP	2,693/420,028	3.01 (2.81-3.22)	1.51 (1.41-1.63)
	Pill	168/29,375	2.85 (2.43-3.36)	1.61 (1.37-1.89)
	FCP	541/77,027	3.51 (3.17-3.89)	1.57 (1.41-1.74)
< 25 kg/m^2	Reference	597/498,975	1.00 (Reference)	1.00 (Reference)
	LCD	221/96,208	1.89 (1.62-2.21)	1.29 (1.10-1.52)
	Exercise	164/116,073	1.40 (1.18-1.67)	1.09 (0.91-1.30)
	LCD & Exercise	516/254,983	1.77 (1.57-2.00)	1.28 (1.13-1.45)
	Fasting	228/114,021	1.89 (1.62-2.20)	1.32 (1.13-1.55)
	CWLP	479/183,887	2.32 (2.05-2.63)	1.40 (1.22-1.60)
	Pill	40/16,551	2.34 (1.70-3.23)	1.45 (1.04-2.00)
	FCP	96/34,924	2.75 (2.21-3.43)	1.58 (1.26-1.98)
25 to < 30 kg/m^2	Reference	433/83,613	1.00 (Reference)	1.00 (Reference)
	LCD	416/66,424	1.16 (1.01-1.33)	1.07 (0.93-1.23)
	Exercise	188/39,796	0.86 (0.72-1.03)	0.85 (0.71-1.01)
	LCD & Exercise	774/146,801	0.97 (0.86-1.09)	0.94 (0.83-1.06)
	Fasting	336/55,942	1.13 (0.98-1.31)	1.04 (0.90-1.21)
	CWLP	1,033/152,525	1.20 (1.07-1.35)	1.14 (1.01-1.29)
	Pill	69/8,681	1.43 (1.11-1.85)	1.32 (1.02-1.71)
	FCP	205/25,687	1.41 (1.19-1.68)	1.25 (1.05-1.49)
≥ 30 kg/m^2	Reference	274/18,088	1.00 (Reference)	1.00 (Reference)
	LCD	433/32,260	0.89 (0.77-1.04)	0.84 (0.72-0.98)
	Exercise	182/14,228	0.81 (0.67-0.98)	0.81 (0.67-0.99)
	LCD & Exercise	647/51,927	0.82 (0.71-0.95)	0.80 (0.70-0.93)
	Fasting	298/21,597	0.89 (0.75-1.05)	0.86 (0.73-1.02)
	CWLP	1,184/83,616	0.92 (0.80-1.05)	0.89 (0.77-1.01)
	Pill	60/4,143	0.91 (0.69-1.21)	0.89 (0.67-1.18)
	FCP	240/16,416	0.94 (0.79-1.13)	0.87 (0.73-1.04)

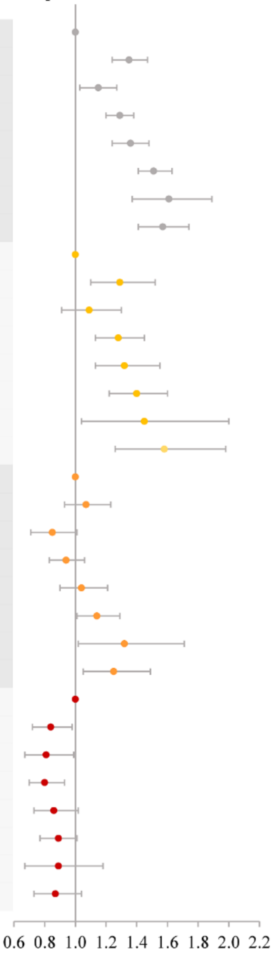

Hazard ratios and 95% confidence intervals were calculated using Cox proportional-hazards model. Multivariable model was adjusted for cohort (Health Professionals Follow-up Study, Nurses' Health Study, or Nurses' Health Study II), age (in months, continuous), ethnicity (white, African American, Asian, or other), baseline body mass index (in kg/m^2, continuous), baseline waist circumference (in cm, continuous), physical activity (in quintiles), television watching (0-1, 2-5, 6-10, 11-20, or >20 hour/week), smoking status (never, past, or current smokers), alcohol intake (0, <5.0, 5.0-9.9, 10.0-14.9, 15.0-29.9, or >30.0 gram/day), hypertension (yes or no), hypercholesterolemia (yes or no), family history of diabetes (yes or no), multivitamin use (yes or no), Alternative Healthy Eating Index score (in quintiles), and total energy intake (in quintiles) before weight loss. **Abbreviations:** BMI, body mass index; CWLP, commercial weight loss program; FCP, select at least two strategies among fasting, CWLP, and pill; LCD, low-calorie diet; kg/m^2, kilogram per square meter.
