# Supplementary material for: Weight loss strategies, weight change, and type 2 diabetes in US health professionals: A cohort study
Source: PLoS Med. 2022 Sep 27;19(9):e1004094. doi: 10.1371/journal.pmed.1004094 (PMC9514663; doi:10.1371/journal.pmed.1004094)
Supplement: S7 Fig — (PDF) [file pmed.1004094.s026.pdf]

S7 Fig. Pooled hazard ratios for association between weight loss strategies and the incidence of type 2 diabetes in participants who were consistently lean/overweight/obese before 1988/1989.

Baseline BMI	Weight Loss Strategies	Cases/ Person-year	Hazard Ratio (95% Confidence Interval) Adjusted for Age	Adjusted for Multiple Covariates
Overall	Reference	1,040/546,348	1.00 (Reference)	1.00 (Reference)
	LCD	782/141,215	2.89 (2.63-3.17)	1.53 (1.39-1.68)
	Exercise	522/160,933	1.77 (1.59-1.97)	1.26 (1.13-1.40)
	LCD & Exercise	1,479/356,056	2.25 (2.07-2.43)	1.44 (1.32-1.56)
	Fasting	742/166,398	2.37 (2.15-2.60)	1.47 (1.33-1.61)
	CWLP	1,947/314,923	3.38 (3.13-3.65)	1.60 (1.48-1.74)
	Pill	153/25,566	3.41 (2.87-4.05)	1.80 (1.52-2.15)
	FCP	458/66,560	3.84 (3.43-4.30)	1.62 (1.44-1.82)
< 25 kg/m^2	Reference	580/488,158	1.00 (Reference)	1.00 (Reference)
	LCD	199/86,733	1.94 (1.65-2.28)	1.37 (1.16-1.61)
	Exercise	159/112,921	1.41 (1.18-1.69)	1.13 (0.94-1.36)
	LCD & Exercise	468/235,397	1.80 (1.59-2.04)	1.34 (1.17-1.52)
	Fasting	209/107,826	1.89 (1.61-2.21)	1.37 (1.16-1.62)
	CWLP	427/165,213	2.40 (2.11-2.73)	1.49 (1.30-1.72)
	Pill	39/15,519	2.51 (1.81-3.48)	1.58 (1.14-2.20)
	FCP	89/33,253	2.73 (2.18-3.43)	1.61 (1.27-2.03)
25 to < 30 kg/m^2	Reference	276/48,073	1.00 (Reference)	1.00 (Reference)
	LCD	259/36,616	1.24 (1.04-1.47)	1.13 (0.95-1.34)
	Exercise	182/35,014	0.94 (0.77-1.14)	0.94 (0.77-1.14)
	LCD & Exercise	522/88,701	1.05 (0.90-1.22)	1.04 (0.89-1.21)
	Fasting	273/43,179	1.13 (0.95-1.34)	1.04 (0.88-1.24)
	CWLP	653/95,485	1.21 (1.04-1.41)	1.17 (1.00-1.36)
	Pill	59/6,682	1.64 (1.23-2.19)	1.47 (1.10-1.97)
	FCP	167/20,088	1.49 (1.22-1.83)	1.31 (1.06-1.60)
≥ 30 kg/m^2	Reference	184/10,117	1.00 (Reference)	1.00 (Reference)
	LCD	324/17,866	1.01 (0.84-1.21)	0.96 (0.80-1.16)
	Exercise	181/12,998	0.80 (0.65-0.99)	0.83 (0.67-1.02)
	LCD & Exercise	489/31,958	0.87 (0.73-1.04)	0.87 (0.73-1.03)
	Fasting	260/15,393	0.95 (0.78-1.15)	0.93 (0.77-1.13)
	CWLP	867/54,225	0.92 (0.78-1.08)	0.89 (0.76-1.05)
	Pill	55/3,365	0.92 (0.68-1.25)	0.92 (0.68-1.25)
	FCP	202/13,218	0.90 (0.73-1.10)	0.83 (0.67-1.02)

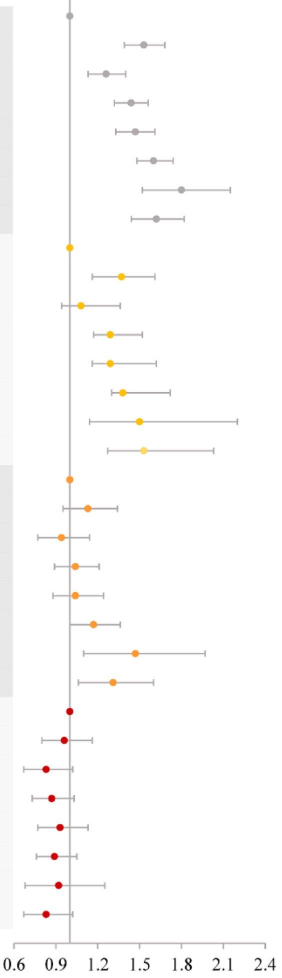

Hazard ratios and 95% confidence intervals were calculated using Cox proportional-hazards model. Multivariable model was adjusted for cohort (Health Professionals Follow-up Study, Nurses' Health Study, or Nurses' Health Study II), age (in months, continuous), ethnicity (white, African American, Asian, or other), baseline body mass index (in kg/m^2, continuous), baseline waist circumference (in cm, continuous), physical activity (in quintiles), television watching (0-1, 2-5, 6-10, 11-20, or >20 hour/week), smoking status (never, past, or current smokers), alcohol intake (0, <5.0, 5.0-9.9, 10.0-14.9, 15.0-29.9, or >30.0 gram/day), hypertension (yes or no), hypercholesterolemia (yes or no), family history of diabetes (yes or no), multivitamin use (yes or no), Alternative Healthy Eating Index score (in quintiles), and total energy intake (in quintiles) before weight loss. **Abbreviations:** BMI, body mass index; CWLP, commercial weight loss program; FCP, select at least two strategies among fasting, CWLP, and pill; LCD, low-calorie diet; kg/m^2, kilogram per square meter.
