# Supplementary material for: Weight loss strategies, weight change, and type 2 diabetes in US health professionals: A cohort study
Source: PLoS Med. 2022 Sep 27;19(9):e1004094. doi: 10.1371/journal.pmed.1004094 (PMC9514663; doi:10.1371/journal.pmed.1004094)
Supplement: S8 Fig — (PDF) [file pmed.1004094.s027.pdf]

S8 Fig. Pooled hazard ratios for association between weight loss strategies and the incidence of type 2 diabetes stratified by maximum BMI before 1992/1993.

Baseline BMI	Weight Loss Strategies	Cases/ Person-year	Hazard Ratio (95% Confidence Interval) Adjusted for Age	Adjusted for Multiple Covariates
Overall	Reference	1,450/600,996	1.00 (Reference)	1.00 (Reference)
	LCD	1,244/195,240	2.56 (2.38-2.77)	1.37 (1.26-1.48)
	Exercise	580/170,199	1.62 (1.47-1.79)	1.15 (1.05-1.27)
	LCD & Exercise	2,162/454,182	2.01 (1.88-2.15)	1.28 (1.20-1.38)
	Fasting	980/191,826	2.26 (2.08-2.45)	1.38 (1.27-1.50)
	CWLP	2,966/420,629	3.05 (2.86-3.25)	1.48 (1.39-1.59)
	Pill	188/29,426	2.98 (2.56-3.47)	1.64 (1.41-1.92)
	FCP	579/77,111	3.51 (3.18-3.87)	1.51 (1.36-1.67)
< 25 kg/m^2	Reference	425/440,176	1.00 (Reference)	1.00 (Reference)
	LCD	107/64,354	1.71 (1.38-2.12)	1.35 (1.08-1.68)
	Exercise	89/87,196	1.41 (1.11-1.78)	1.27 (1.00-1.62)
	LCD & Exercise	245/172,409	1.64 (1.39-1.92)	1.40 (1.18-1.65)
	Fasting	110/80,674	1.81 (1.46-2.24)	1.45 (1.16-1.80)
	CWLP	174/99,295	2.09 (1.74-2.51)	1.58 (1.30-1.92)
	Pill	18/10,171	2.44 (1.52-3.92)	1.97 (1.22-3.18)
	FCP	33/20,497	2.26 (1.58-3.24)	1.71 (1.19-2.46)
25 to < 30 kg/m^2	Reference	582/127,628	1.00 (Reference)	1.00 (Reference)
	LCD	395/77,393	1.11 (0.97-1.26)	1.03 (0.90-1.17)
	Exercise	207/57,327	0.88 (0.75-1.03)	0.89 (0.75-1.05)
	LCD & Exercise	810/186,257	0.98 (0.88-1.09)	0.98 (0.87-1.09)
	Fasting	360/71,645	1.19 (1.04-1.35)	1.11 (0.97-1.27)
	CWLP	834/174,525	1.07 (0.96-1.20)	1.06 (0.94-1.19)
	Pill	52/11,242	1.10 (0.83-1.47)	1.03 (0.77-1.38)
	FCP	149/26,981	1.32 (1.09-1.59)	1.22 (1.01-1.48)
≥ 30 kg/m^2	Reference	443/33,192	1.00 (Reference)	1.00 (Reference)
	LCD	742/53,493	1.04 (0.92-1.17)	0.95 (0.84-1.07)
	Exercise	284/25,675	0.84 (0.72-0.98)	0.82 (0.70-0.95)
	LCD & Exercise	1,107/95,516	0.87 (0.78-0.98)	0.84 (0.75-0.94)
	Fasting	510/39,507	0.98 (0.86-1.12)	0.91 (0.80-1.04)
	CWLP	1,958/146,809	0.99 (0.90-1.10)	0.94 (0.85-1.05)
	Pill	118/8,013	1.10 (0.90-1.35)	1.08 (0.88-1.33)
	FCP	397/29,633	1.01 (0.88-1.16)	0.92 (0.80-1.06)

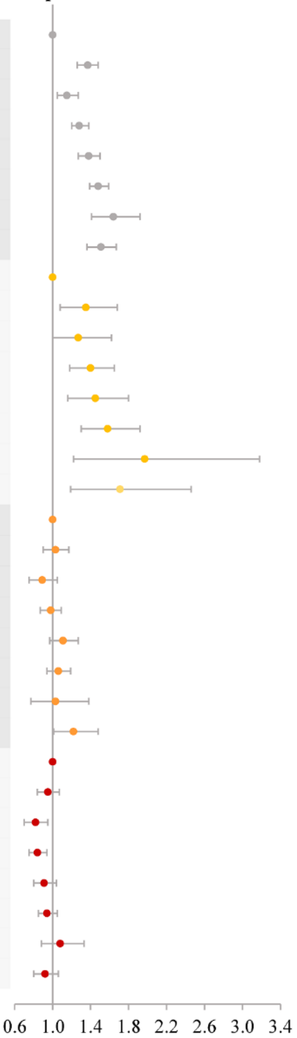

Hazard ratios and 95% confidence intervals were calculated using Cox proportional-hazards model. Multivariable model was adjusted for cohort (Health Professionals Follow-up Study, Nurses' Health Study, or Nurses' Health Study II), age (in months, continuous), ethnicity (white, African American, Asian, or other), baseline body mass index (in kg/m^2, continuous), baseline waist circumference (in cm, continuous), physical activity (in quintiles), television watching (0-1, 2-5, 6-10, 11-20, or >20 hour/week), smoking status (never, past, or current smokers), alcohol intake (0, <5.0, 5.0-9.9, 10.0-14.9, 15.0-29.9, or >30.0 gram/day), hypertension (yes or no), hypercholesterolemia (yes or no), family history of diabetes (yes or no), multivitamin use (yes or no), Alternative Healthy Eating Index score (in quintiles), and total energy intake (in quintiles) before weight loss. **Abbreviations:** BMI, body mass index; CWLP, commercial weight loss program; FCP, select at least two strategies among fasting, CWLP, and pill; LCD, low-calorie diet; kg/m^2, kilogram per square meter.
