# Supplementary material for: Weight loss strategies, weight change, and type 2 diabetes in US health professionals: A cohort study
Source: PLoS Med. 2022 Sep 27;19(9):e1004094. doi: 10.1371/journal.pmed.1004094 (PMC9514663; doi:10.1371/journal.pmed.1004094)
Supplement: S9 Fig — (PDF) [file pmed.1004094.s028.pdf]

S9 Fig. Pooled hazard ratios for association between weight loss strategies and the incidence of type 2 diabetes (participants who skipped the weight loss strategy question were included into the reference group).

Baseline BMI	Weight Loss Strategies	Cases/ Person-year	Hazard Ratio (95% Confidence Interval) Adjusted for Age	Adjusted for Multiple Covariates
Overall	Reference	1,657/669,366	1.00 (Reference)	1.00 (Reference)
	LCD	1,242/195,252	2.51 (2.33-2.71)	1.34 (1.25-1.45)
	Exercise	580/170,199	1.60 (1.45-1.76)	1.14 (1.03-1.26)
	LCD & Exercise	2,162/454,206	1.98 (1.85-2.11)	1.27 (1.19-1.36)
	Fasting	979/191,817	2.22 (2.05-2.40)	1.36 (1.25-1.47)
	CWLP	2,966/420,526	3.00 (2.82-3.20)	1.47 (1.38-1.57)
	Pill	188/29,426	2.94 (2.52-3.43)	1.62 (1.39-1.89)
	FCP	579/77,111	3.46 (3.14-3.81)	1.50 (1.35-1.65)
< 25 kg/m^2	Reference	737/552,787	1.00 (Reference)	1.00 (Reference)
	LCD	236/96,259	1.85 (1.59-2.14)	1.27 (1.09-1.48)
	Exercise	170/116,090	1.38 (1.16-1.63)	1.08 (0.90-1.28)
	LCD & Exercise	553/255,080	1.77 (1.58-1.98)	1.29 (1.15-1.45)
	Fasting	236/114,041	1.84 (1.58-2.13)	1.29 (1.11-1.51)
	CWLP	496/183,936	2.27 (2.01-2.55)	1.38 (1.21-1.57)
	Pill	44/16,562	2.43 (1.79-3.30)	1.50 (1.10-2.05)
	FCP	96/34,924	2.63 (2.12-3.27)	1.53 (1.22-1.91)
25 to < 30 kg/m^2	Reference	563/96,180	1.00 (Reference)	1.00 (Reference)
	LCD	474/66,547	1.20 (1.06-1.36)	1.10 (0.97-1.25)
	Exercise	209/39,844	0.91 (0.77-1.07)	0.90 (0.77-1.06)
	LCD & Exercise	856/146,973	0.99 (0.89-1.10)	0.96 (0.86-1.08)
	Fasting	387/56,056	1.20 (1.05-1.37)	1.10 (0.97-1.26)
	CWLP	1,103/152,690	1.22 (1.09-1.35)	1.16 (1.04-1.29)
	Pill	77/8,702	1.54 (1.21-1.96)	1.41 (1.10-1.80)
	FCP	218/25,714	1.45 (1.23-1.71)	1.28 (1.09-1.51)
≥ 30 kg/m^2	Reference	357/20,400	1.00 (Reference)	1.00 (Reference)
	LCD	532/32,446	0.94 (0.82-1.07)	0.87 (0.76-1.00)
	Exercise	201/14,264	0.81 (0.68-0.96)	0.81 (0.68-0.97)
	LCD & Exercise	753/52,154	0.83 (0.73-0.94)	0.81 (0.71-0.92)
	Fasting	356/21,720	0.93 (0.80-1.08)	0.89 (0.77-1.03)
	CWLP	1,367/83,901	0.93 (0.83-1.05)	0.89 (0.79-1.01)
	Pill	67/4,162	0.91 (0.70-1.19)	0.89 (0.68-1.16)
	FCP	265/16,473	0.92 (0.78-1.08)	0.84 (0.71-0.99)

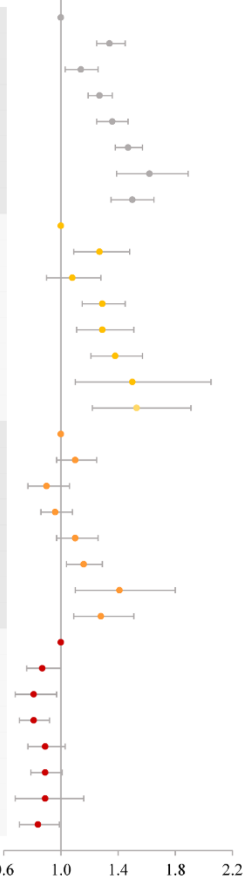

Hazard ratios and 95% confidence intervals (CIs) were calculated using Cox proportional-hazards model. Multivariable model was adjusted for cohort (Health Professionals Follow-up Study, Nurses' Health Study, or Nurses' Health Study II), age (in months, continuous), ethnicity (white, African American, Asian, or other), baseline body mass index (in kg/m^2, continuous), baseline waist circumference (in cm, continuous), physical activity (in quintiles), television watching (0-1, 2-5, 6-10, 11-20, or >20 hour/week), smoking status (never, past, or current smokers), alcohol intake (0, <5.0, 5.0-9.9, 10.0-14.9, 15.0-29.9, or >30.0 gram/day), hypertension (yes or no), hypercholesterolemia (yes or no), family history of diabetes (yes or no), multivitamin use (yes or no), Alternative Healthy Eating Index score (in quintiles), and total energy intake (in quintiles) before weight loss. **Abbreviations:** BMI, body mass index; CWLP, commercial weight loss program; FCP, select at least two strategies among fasting, CWLP, and pill; LCD, low-calorie diet; kg/m^2, kilogram per square meter.
