# Supplementary material for: Weight loss strategies, weight change, and type 2 diabetes in US health professionals: A cohort study
Source: PLoS Med. 2022 Sep 27;19(9):e1004094. doi: 10.1371/journal.pmed.1004094 (PMC9514663; doi:10.1371/journal.pmed.1004094)
Supplement: S11 Fig — (PDF) [file pmed.1004094.s030.pdf]

S11 Fig. Weight loss strategies and absolute weight change percentages since baseline stratified by baseline abdominal obesity status.

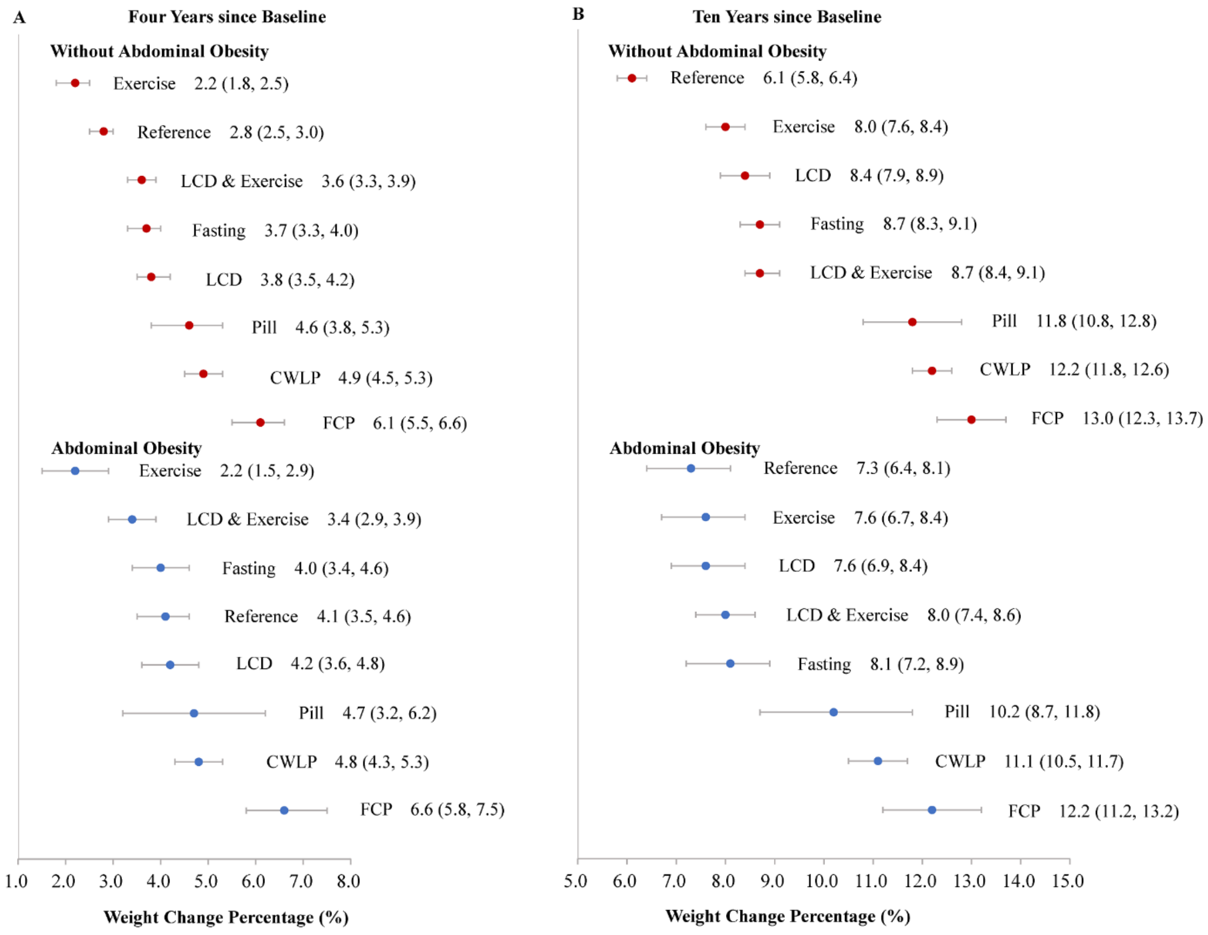

Least squares means (95% conference intervals) for weight change percentage were calculated using generalized estimating equation, with adjustment for cohort (Health Professionals Follow-up Study, Nurses' Health Study, or Nurses' Health Study II), age (in month, continuous), ethnicity (white, African American, Asian, or other), baseline body weight (in kilogram, continuous), baseline waist circumference (in cm, continuous), physical activity (in quintiles), television watching (0-1, 2-5, 6-10, 11-20, or >20 hour/week), smoking status (never, past, or current smokers), alcohol intake (0, <5.0, 5.0-9.9, 10.0-14.9, 15.0-29.9, or >30.0 gram/day), hypertension (yes or no), hypercholesterolemia (yes or no), family history of diabetes (yes or no), multivitamin use (yes or no), Alternative Healthy Eating Index score (in quintiles), and total energy intake (in quintiles) before weight loss. **Abbreviations:** LCD, low-calorie diet; CWLP, commercial weight loss program; FCP, select at least two strategies among fasting, CWLP, and pill.
