# Supplementary material for: Weight loss strategies, weight change, and type 2 diabetes in US health professionals: A cohort study
Source: PLoS Med. 2022 Sep 27;19(9):e1004094. doi: 10.1371/journal.pmed.1004094 (PMC9514663; doi:10.1371/journal.pmed.1004094)
Supplement: S12 Fig — (PDF) [file pmed.1004094.s031.pdf]

S12 Fig. Pooled hazard ratios for association between weight loss strategies and the incidence of type 2 diabetes stratified by baseline abdominal obesity status.

Baseline Status	Weight Loss Strategies	Cases/Person-year	Hazard Ratio (95% Confidence Interval) Adjusted for Age	Adjusted for Multiple Covariates
Without abdominal obesity	Reference	987/553,456	1.00 (ref.)	1.00 (ref.)
	LCD	554/142,531	2.09 (1.88-2.32) *	1.25 (1.11-1.40)*
	Exercise	293/141,212	1.48 (1.30-1.69)*	1.02 (0.88-1.18)
	LCD & Exercise	1,131/357,519	1.84 (1.69-2.00)*	1.15 (1.05-1.26)*
	Fasting	485/150,487	2.02 (1.81-2.26)*	1.30 (1.15-1.46)*
	CWLP	1,242/283,505	2.65 (2.43-2.88)*	1.36 (1.23-1.50)*
	Pill	92/21,486	2.93 (2.36-3.63)*	1.69 (1.34-2.12)*
	FCP	215/48,767	3.03 (2.61-3.52)*	1.49 (1.28-1.74)*
Abdominal obesity	Reference	463/47,540	1.00 (ref.)	1.00 (ref.)
	LCD	690/52,710	1.33 (1.18-1.50)*	1.03 (0.90-1.18)
	Exercise	287/28,987	1.10 (0.95-1.28)	0.94 (0.80-1.10)
	LCD & Exercise	1,031/96,663	1.10 (0.98-1.23)	0.96 (0.85-1.08)
	Fasting	495/41,339	1.26 (1.11-1.43)*	1.02 (0.88-1.17)
	CWLP	1,724/137,124	1.31 (1.18-1.45)*	1.06 (0.94-1.18)
	Pill	96/7,940	1.33 (1.07-1.67)*	1.09 (0.87-1.38)
	FCP	364/28,344	1.40 1.22-1.61)*	1.05 (0.91-1.22)

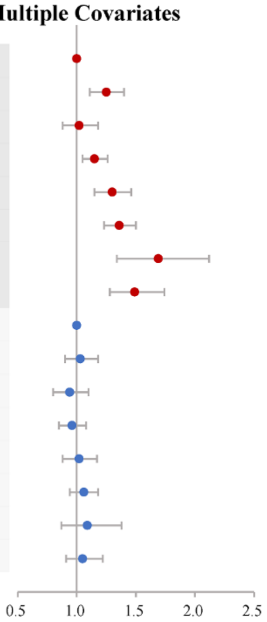

Hazard ratios and 95% confidence intervals (CIs) were calculated using Cox proportional-hazards model. Multivariable model was adjusted for cohort (Health Professionals Follow-up Study, Nurses' Health Study, or Nurses' Health Study II), age (in months, continuous), ethnicity (white, African American, Asian, or other), baseline body mass index (in kg/m^2, continuous), baseline waist circumference (in cm, continuous), physical activity (in quintiles), television watching (0-1, 2-5, 6-10, 11-20, or >20 hour/week), smoking status (never, past, or current smokers), alcohol intake (0, <5.0, 5.0-9.9, 10.0-14.9, 15.0-29.9, or >30.0 gram/day), hypertension (yes or no), hypercholesterolemia (yes or no), family history of diabetes (yes or no), multivitamin use (yes or no), Alternative Healthy Eating Index score (in quintiles), and total energy intake (in quintiles) before weight loss. *P* for interaction for overall and individual weight loss strategies were less than 0.001. The asterisk indicated that the false discovery rate was less than 0.05. **Abbreviations:** CWLP, commercial weight loss program; FCP, select at least two strategies among fasting, CWLP, and pill; LCD, low-calorie diet.
